# *Muddy*, *muddled*, or *muffled*? Understanding the perception of audio quality in music by hearing aid users

**DOI:** 10.3389/fpsyg.2024.1310176

**Published:** 2024-02-21

**Authors:** Scott Bannister, Alinka E. Greasley, Trevor J. Cox, Michael A. Akeroyd, Jon Barker, Bruno Fazenda, Jennifer Firth, Simone N. Graetzer, Gerardo Roa Dabike, Rebecca R. Vos, William M. Whitmer

**Affiliations:** ^1^School of Music, University of Leeds, Leeds, United Kingdom; ^2^Acoustics Research Centre, University of Salford, Salford, United Kingdom; ^3^School of Medicine, University of Nottingham, Nottingham, United Kingdom; ^4^Department of Computer Science, University of Sheffield, Sheffield, United Kingdom

**Keywords:** music, hearing loss, audio quality evaluation, perception, hearing aids

## Abstract

**Introduction:**

Previous work on audio quality evaluation has demonstrated a developing convergence of the key perceptual attributes underlying judgments of quality, such as timbral, spatial and technical attributes. However, across existing research there remains a limited understanding of the crucial perceptual attributes that inform audio quality evaluation for people with hearing loss, and those who use hearing aids. This is especially the case with music, given the unique problems it presents in contrast to human speech.

**Method:**

This paper presents a sensory evaluation study utilising descriptive analysis methods, in which a panel of hearing aid users collaborated, through consensus, to identify the most important perceptual attributes of music audio quality and developed a series of rating scales for future listening tests. Participants (*N* = 12), with a hearing loss ranging from mild to severe, first completed an online elicitation task, providing single-word terms to describe the audio quality of original and processed music samples; this was completed twice by each participant, once with hearing aids, and once without. Participants were then guided in discussing these raw terms across three focus groups, in which they reduced the term space, identified important perceptual groupings of terms, and developed perceptual attributes from these groups (including rating scales and definitions for each).

**Results:**

Findings show that there were seven key perceptual dimensions underlying music audio quality (*clarity*, *harshness*, *distortion*, *spaciousness*, *treble strength*, *middle strength*, and *bass strength*), alongside a music audio quality attribute and possible alternative frequency balance attributes.

**Discussion:**

We outline how these perceptual attributes align with extant literature, how attribute rating instruments might be used in future work, and the importance of better understanding the music listening difficulties of people with varied profiles of hearing loss.

## 1 Introduction

Many people spend a substantial amount of time engaging with music, including through music-making (Clift and Hancox, 2001; Kokotsaki and Hallam, 2007), attendance at events (Brown and Knox, 2017), and music listening in everyday life (Lamont et al., 2016). There are a plethora of benefits of musical engagement: music can help to regulate mood and emotion (Saarikallio, 2011; Schafer et al., 2013; Taruffi and Koelsch, 2014), facilitate social bonding and belonging (Kirschner and Tomasello, 2009; D’Ausilio et al., 2015; Groarke et al., 2022), support the construction of self and identity (DeNora, 2000; Lonsdale, 2021), and give pleasure (Zatorre and Salimpoor, 2013); indeed, music can be an exceptional form of therapy (Grocke and Wigram, 2006; Hurt-Thaut, 2016). As such, barriers to musical engagement can, for some, be significantly problematic for general wellbeing.

One potential barrier to musical engagement is hearing loss. Hearing loss affects over 1.5 billion people globally and is estimated to increase to 2.5 billion by 2050 (World Health Organization, 2021). This may be due to ageing populations, but with portable technologies that enable pervasive music listening, hearing loss is also apparent in younger populations (Jiang et al., 2016). Musicians are at risk of noise-induced hearing loss due to prolonged exposure to loud sounds (Couth et al., 2019; Greasley et al., 2020a). Many people, including musicians, can also develop age-related hearing loss, or presbycusis. Presbycusis might often be characterised by elevated hearing thresholds for higher frequency sounds, and in some cases increased sensitivity to rapid increases in loudness (i.e., loudness recruitment). However, it is important to acknowledge the scope and variability of hearing loss causes, types, and experiences.

Hearing aid technologies have been developed to address issues commonly found in hearing loss, especially regarding speech intelligibility and quality. Hearing aids often utilize digital signal processing strategies (e.g., dynamic range compression, beamforming, noise reduction) that can enhance incoming sound signals for listeners. Whilst many of these strategies work well for speech, they are less effective for music due to spectrotemporal differences between speech and music. Chasin and Russo (2004) note four key differences: (1) there is no long-term spectrum across different pieces of music, and is spectrally variable in comparison to speech; (2) perception of important elements in musical sounds depends on diverse spectrotemporal properties varying across instruments, in contrast to more consistent properties of speech; (3) music has a greater range of intensity compared to speech, and (4) relatedly, music can have higher crest factors (i.e., peak energy value of signal divided by the root-mean-square energy value of signal) than speech. Given these differences, several hearing aid manufacturers have developed music programs for their devices to enhance the experience of listening to music, though evidence for their efficacy is mixed (Madsen and Moore, 2014; Looi et al., 2019; Vaisberg et al., 2019). Although hearing aids do help in music listening scenarios, there remain numerous difficulties that users experience (Greasley et al., 2020b), including pitch perception issues, problems with live music contexts, volume and dynamics, feedback, distortion, and hearing lyrics. In other work by Looi et al. (2019), hearing aids were reported to affect the melodic quality of music for listeners with moderate to severe levels of hearing loss.

Music listening difficulties encountered through hearing loss and use of hearing aids may be considered from the perspective of audio quality, as distinct from the qualities of the ‘music’ itself (e.g., the hierarchical structuring of rhythm, melody, harmony, and themes). There is substantial literature that explores the evaluation of audio quality (Gabrielsson, 1979; Toole, 1985; Zacharov and Koivuniemi, 2001; Berg and Rumsey, 2006; Wilson and Fazenda, 2016; Woodcock et al., 2018; Pike, 2019), ranging from holistic measures of quality (e.g., good vs. bad), to investigations of perceptual attributes that comprise judgments of audio quality. However, research on music listening experiences of hearing-impaired individuals and hearing aid users remains scarce. It is crucial to understand the key perceptual attributes of audio quality for these listeners, as this provides a perceptual foundation for developments of signal processing algorithms for music in hearing aid technology, and for future innovations in intelligent music (re-)mixing and object-based audio approaches. The current study aims to contribute to this foundation.

### 1.1 Audio quality evaluation

Hearing aids, and the signal processing strategies they employ, have been investigated in the context of audio quality; such work often focusses on speech intelligibility or quality, but can also include music excerpts. Studies in this area have contributed significantly to understanding how non-linear (e.g., dynamic range compression) and linear (e.g., bandwidth filtering) signal processing approaches can affect perceived audio quality, a variable mostly considered at a holistic level of resolution (e.g., good or bad), and often captured through stimulus comparison and preference approaches (e.g., better or worse), with some exceptions (see Balfour and Hawkins, 1992; Souza et al., 2007; Vaisberg et al., 2021a). In terms of compression, studies have shown that for music, both normal-hearing and hearing-impaired listeners may prefer compression ratios closer to linear amplification (van Buuren et al., 1999; Hansen, 2002; Arehart et al., 2011; Kirchberger and Russo, 2016), prefer wide dynamic range compression over clipping and limiting (Souza et al., 2007), and prefer slow compression speeds (Moore et al., 2011; Croghan et al., 2014). In terms of how compression is applied across different frequency bands, research demonstrates important inter-individual variation in terms of what is preferred (Lunner et al., 1997; Ricketts et al., 2008; Moore et al., 2011), perhaps depending on variations across hearing profiles of listeners and the stimuli used. In terms of spectral filtering and frequency responses, studies suggest that people with hearing loss may prefer large bandwidths (Punch, 1978; Ricketts et al., 2008; Arehart et al., 2011), although this was less clear in earlier work by Franks (1982) and may depend on the severity of hearing loss (Brennan et al., 2014). Listeners may also link processing strategies that emphasise lower frequencies to better audio quality (Franks, 1982; Arehart et al., 2011; Vaisberg et al., 2021b). Finally, in work involving normal-hearing listeners, Moore and Tan (2003) linked various spectral filters to ratings of naturalness of audio; for example, increased spectral ripple depth, rate and range resulted in lower ratings of naturalness, with comparable results found for wider range spectral tilt. Beyond these signal processing strategies, research on sound quality involving music has also focused on other variables, including different prescription fittings for hearing aids (Moore and Sek, 2013, 2016; Moore et al., 2016), frequency compression strategies (Parsa et al., 2013; Uys and Latzel, 2015; Kirchberger and Russo, 2016), and reverberation (Reinhart and Souza, 2018). Given the heterogeneity of music signal properties compared to speech, many of the above findings are likely sensitive to variations across music styles or genres (Arehart et al., 2011; Higgins et al., 2012); however, most work in this field utilizes a small corpus of music excerpts that does not encapsulate the diversity of style and genre.

As noted, it is rare that experimental approaches to hearing aid processing and music assess audio quality beyond a holistic rating of quality or preference, limiting an understanding of the perceptual dimensions of music audio quality that underpin judgments and experience. Those few studies that do explore audio quality features often utilize a series of scales developed by Gabrielsson (1979), Gabrielsson and Sjogren (1979), and Gabrielsson et al. (1988). These scales were initially developed by collating roughly 200 adjectives from various sources (Gabrielsson, 1979, p. 160), and asking sound engineers, audiologists, and people with hearing loss to judge the suitability of each adjective in describing how loudspeakers, headphones or hearing aids may sound. Through this process and other reductions of the adjective space, 30–50 adjectives were taken forward (depending on experiment), and across experiments utilizing adjective ratings, similarity ratings, and free responses, it was proposed that results reflect seven perceptual dimensions of sound quality: *fullness*, *loudness, brightness, softness, nearness, spaciousness,* and *clarity*.

Work on understanding the perceptual attributes that underpin evaluations of audio quality has continued to develop (for a review, see Pike, 2019). The perceptual structure of audio quality has been explored in the context of spatial sound reproduction (Koivuniemi and Zacharov, 2001; Lorho, 2005; Berg and Rumsey, 2006), multichannel and binaural reproduced sound (Nakayama et al., 1971; Guastavino and Katz, 2004; Rumsey et al., 2005; Choisel and Wickelmaier, 2006, 2007; Pike, 2019), room acoustics (Lokki et al., 2011; Wankling et al., 2012), object-based audio remixing of television broadcasts (Woodcock et al., 2018), and the comparison of hearing aid models (Legarth et al., 2014). Given the subjective perceptual space being investigated across this work, alongside diverse methodological approaches, a substantial variability can be found in the attributes that are generated. Importantly however, there is some convergence of audio quality attributes across research, with key perceptual constructs proposed. For instance, Letowski (1989) proposed the MURAL model to encapsulate perceptual dimensions of sound quality assessment, which mostly fell into two main categories of *timbre* (e.g., brightness, sharpness, coloration) and *spaciousness* (e.g., perspective, ambience, panorama), though some attributes were positioned in both groups (e.g., presence, blend, clarity). Berg and Rumsey (2003) also proposed a model of audio quality that encompassed perceptual attributes in terms of *timbral, spatial*, *technical,* and *miscellaneous* categories. More recently, Le Bagousse et al. (2014) selected attributes from extant literature and asked audio experts to evaluate their suitability and relevance for audio quality assessment; experiment panelists assessed similarity between attributes in one experiment and performed a free choice grouping of attributes in the other. Results suggest that the perceptual attribute space of audio quality can be divided into four categories: *Timbral* (e.g., brightness, tone colour, richness)*, spatial* (e.g., depth, reverberation, width, distance)*, defects* (e.g., noise, distortion, hiss, hum), and *quality* (e.g., realism, fidelity, dynamics, stability). These categories are further reflected in a recent ‘sound wheel’ lexicon of audio quality developed by Pedersen and Zacharov (2015), utilising attributes from existing literature and working with expert panels to identify mappings and clusters of attributes. This model notes *timbre, spatial* and *artefacts* as three overarching perceptual groupings, reflecting the work by Berg and Rumsey (2003) and Le Bagousse et al. (2014).

The above work is crucial to understanding audio quality evaluation, especially the *why* of judgments by listeners (Pike, 2019). However, in the context of music listening and hearing loss, previous studies and conceptual models are limited. Most audio quality evaluation studies rarely consider the perceptual experiences of people with hearing loss, and those who use hearing aids. An exception may be the scales developed by Gabrielsson and Sjogren (1979), with hearing-impaired people contributing to the initial reduction of the attribute space. However, hearing-impaired listeners were not often involved in the exploration of these attributes in different modes of sound reproduction, and this includes hearing aid sound reproduction (Gabrielsson, 1979). Indeed, it has been suggested that in the case of hearing aid users, the seven perceptual dimensions developed in Gabrielsson’s work are limited in terms of reliability (Narendran and Humes, 2003). Furthermore, across the few investigations that involve listeners with hearing loss, participants are often presented with a perceptual attribute space or list of terms to work with; as these attributes are mostly informed by and derived from extant literature that focusses on ‘normal’ hearing experiences, it is plausible that they are not fully applicable to, or sufficient to describe, the lived experiences of hearing-impaired participants. Given existing research and these limitations, it is imperative to develop a listener-driven understanding of the perceptual experiences of music audio quality, from the perspectives of those with hearing loss and those who use hearing aids.

### 1.2 Descriptive analysis

Methods from sensory evaluation research are well-suited to developing an understanding of music audio quality perception in hearing aid users. These methods are used to elicit and analyze responses to stimuli linked to primary sensory perceptions, like olfactory, gustatory, and auditory perception (Stone et al., 2012). Historically, they have been developed in the study of consumer experiences of food and other products (Lawless and Heymann, 2010), but have seen effective usage in studies of audio and sound perception (Zacharov, 2019).

This current study utilizes the descriptive analysis (DA) method, a consensus vocabulary approach in which descriptive terms are first elicited across a panel of participants using various music samples. Subsequently, through focus groups, this *sensory panel* consensually identifies and agrees on the important perceptual attributes and constructs a method of measuring these. The present work adapts a generic DA procedure proposed by Lawless and Heymann (2010), which is outlined as follows for the current context: (1) selection of stimuli for eliciting perceptual terms; (2) recruitment and screening of participants; (3) eliciting terms from participants individually using a stimulus set; (4) focus groups to identify important perceptual attributes, and develop measurement tools; (5) check participant understanding and usability of attribute definitions and measurement tools. This inductive approach was preferred, given the limited understanding of music audio quality as perceived by hearing aid users, and the diverse experiences of individuals within and across levels of hearing loss.

### 1.3 Aims

The main aim of this sensory evaluation study was to develop an understanding of the important perceptual attributes of music audio quality for hearing aid users. By using music samples to elicit perceptual terms from listeners and running focus groups for listeners to reach a consensus on the most important perceptual attributes, the work also aimed to produce perceptual metrics for use in future behavioural and experimental work.

## 2 Overall methodology

The remainder of this paper firstly outlines the overall study methodology, before focussing on the design, procedures, and results of the specific stages of the sensory panel work, which are *individual elicitation, three focus groups,* and *follow-up*. As these stages were iterative and developed from results of preceding stages, the procedures and results are outlined in turn.

The study received ethical approval from the Institutional Research Ethics Committee (approval number: FAHC 21-125) and was carried out from November 2022 to April 2023. The individual elicitation and follow-up stages were performed online via Qualtrics. Each focus group lasted 4 h and was split into a morning and afternoon session of 2 h each. Focus groups took place in a meeting room in the School of Music, University of Leeds. Researchers facilitating the focus groups were careful in ensuring that the experiences of all participants were taken into consideration, and that each participant had a voice in the discussion; consequently, there were no dominant participants in these sessions.

### 2.1 Participants

Twelve hearing aid users were recruited through an existing network of participants developed across the Hearing Aids for Music research project,^1^ and through professional networks. The main inclusion criteria were that participants were bilateral hearing aid users and had a hearing loss within the range of mild through to severe; given the tasks and activities involved for participants in the study, a decision was made not to involve people with a profound level of hearing loss. The panel was comprised of 6 males and 6 females, with a mean age of 60.80 (SD = 19.03; range = 38–85; two missing values).

Hearing loss categorization followed the WHO-proposed four frequency average (Humes, 2019), with one participant characterised as having ‘no hearing impairment’ (according to the WHO better ear averages; however, this participant was a bilateral hearing aid user, and had a high-frequency hearing loss), two with mild loss, five with moderate loss, three with moderately severe loss, and one with severe loss. One participant had an asymmetrical hearing loss, with a severe loss in the right ear and a mild loss in the left. Hearing loss profiles were measured as part of the present study via pure tone audiometry listening tests performed by a trained researcher (second author), using an Interacoustics AC40 Clinical Audiometer 2018 model. Audiometry data and participant details are provided in Table 1.

**Table 1:** Participant details, and audiometry data summary.

Participant	Age	Gender	Hearing loss duration	Hearing aid type	Hearing aid use duration	Music program	Music program use	PTA right ear average (dB)	PTA left ear average (dB)	Overall hearing loss level (better ear average)
1	–	Female	5–10 years	BTE	5+ years	Yes	Often	52.50	60.00	Moderately severe
2	–	Male	10–20 years	BTE	5+ years	No	NA	32.50	43.75	Mild
3	84	Female	20+ years	BTE	5+ years	Yes	Sometimes	71.25	66.25	Severe
4	38	Male	20+ years	BTE	5+ years	No	NA	45.00	42.50	Moderate
5	64	Male	5–10 years	BTE	3–4 years	Yes	Sometimes	35.00	37.50	Moderate
6	85	Male	20+ years	BTE	5+ years	Yes	Occasionally	63.75	65.00	Moderately severe
7	80	Female	10–20 years	BTE	5+ years	Yes	Never	53.75	46.25	Moderate
8	52	Male	5–10 years	BTE	5+ years	No	NA	70.00	22.50	Mild
9	38	Male	5–10 years	BTE	6–12 months	No	NA	21.25	18.75	No impairment
10	75	Female	20+ years	BTE	5+ years	Yes	Often	53.75	58.75	Moderately severe
11	46	Female	5–10 years	BTE	5+ years	No	NA	38.75	38.75	Moderate
12	46	Female	20+ years	BTE	5+ years	No	NA	47.50	45.00	Moderate

Frequencies tested in the PTA were [250 Hz, 500 Hz, 1 kHz, 2 kHz, 3 kHz, 4 kHz, 6 kHz, 8 kHz, and 12.5 kHz]. Better ear average was calculated across hearing threshold values at 500 Hz, 1 kHz, 2 kHz and 4 kHz, with hearing loss levels (based on average) derived from the WHO 4-frequency classification (Humes, 2019). BTE, behind the ear; PTA, pure tone audiometry.

Participants completed a background questionnaire (see Supplementary material) that collected information on hearing loss and hearing aid history. Musical background was captured via the 18-item general sophistication factor of the Goldsmith’s Musical Sophistication Index (GOLD-MSI; Müllensiefen et al., 2014), with a factor score ranging from 18 to 126 (higher values indicating higher sophistication); the mean score for the sample was 76.18 (SD = 19.52; one missing value), in comparison to a population norm score of 81.58 (Müllensiefen et al., 2014). Musical preferences were explored via the Revised Short Test of Musical Preferences (STOMP-R; Rentfrow et al., 2011), rating preference for 21 music genres through 1–7 Likert-type scales; these reflect higher order preference dimensions of *mellow* (e.g., world, new age, sample M = 4.69, SD = 0.94)*, unpretentious* (e.g., pop, country, M = 4.87, SD = 0.83; one missing value)*, sophisticated* (e.g., classical, jazz, M = 5.44, SD = 0.64; one missing value)*, intense* (e.g., rock, punk, M = 4.43, SD = 1.36; one missing value), and *contemporary* (e.g., rap, soul, M = 4.47, SD = 1.24; one missing value).

### 2.2 Music samples and processing

To create music samples for the individual elicitation task, 10 music recordings were taken from the MedleyDB 2.0 database (Bittner et al., 2016); this database contains 74 multitrack recordings (providing access to raw audio tracks and audio mixing stems). Recordings were selected to represent several music styles (e.g., classical, pop, rock, jazz, opera), and to contain instrumental music and music with lyrics (five recordings for each). For each recording an excerpt of between 12 and 17 s (mean duration = 14.50 s) was extracted, and these samples were selected subjectively to encapsulate various spectral balance characteristics, instrumental density or complexity, and dynamic changes. This variability was important for generating diverse listening experiences and a broader representative perceptual term space. The 10 music samples are provided in the research data release on FigShare (see data availability statement).

To further capture diverse perceptual experiences, music samples were subject to signal processing or mixing strategies, informed by existing literature. For each sample, a *compressed*, *band-pass*, and *car noise* version was created. For compression limiting, samples were passed through the ‘dRC’ compressor function in MATLAB (The MathWorks Inc., 2022), with a 10:1 ratio, an attack time of 5 ms, and release time of 70 ms. For bandpass filtering, samples were passed through the ‘bandpass’ filter function in MATLAB, with a low cutoff of 300 Hz, high cutoff of 5,000 Hz, and steepness of cutoff set to 0.95. These processing strategies were adapted from work by Arehart et al. (2010, 2011) that explored signal processing strategies associated with hearing aid technology, and how these affect speech quality and music audio quality. Furthermore, these processing strategies were extreme in their effects on music to ensure that a comprehensive perceptual term space was captured. Finally, for the car noise versions, samples were first convolved with a car room impulse response taken from the ELOSPHERES binaural room impulse response database (Hilkhuysen, 2021); this convolution was achieved through the Logic Pro Space Designer convolution reverb plug-in (with a direct signal level of 0 dB and reverberation signal level of –10 dB). Following this, a recording of car noise was added to the convolved music recording, taken from the In-Vehicle Noise Dataset (Magic Data Technology, 2022), with a signal-to-noise ratio of +5 dB (±0.05 dB). This ratio was determined to provide an appropriate balance of noise interference that would impact perceptual experience, without the music signal appearing to be obfuscated by a superimposed and separate layer of noise. The broad convolution approach was used to give a sense of the music being played in a car space. This processing condition was included to capture a common problematic listening scenario for hearing aid users, and to link the development of perceptual audio quality measures to scenarios addressed in an ongoing machine learning challenge.

As a final exploratory approach, new samples were created by re-mixing audio stems, in line with technological developments and the potential of object-based audio formats (Ward and Shirley, 2019). As there is currently little work that establishes a rationale for what should be re-mixed and how, a decision was taken to categorize audio stems for a music recording into vocals, drums, bass and other, following prevalent distinctions in audio source separation research (Liutkus et al., 2017). Only the five recordings containing lyrics were re-mixed, to maximise the total stems that could be remixed. Next, it was decided that new samples would be generated by changes to a single stem category of the mix, restricted to +6 dB or –6 dB in level; this meant that for any music recording with lyrics (with one exception, as one sample contained no bass stem), there would be 8 new samples generated (i.e., ±vocals, drums, bass, or other).

Overall, including original versions, 78 samples were produced. However, to keep the individual elicitation task to a reasonable length and duration, 27 of these samples were used, with each sample listened to twice (once aided, and once unaided). Table 2 outlines the samples used in the individual elicitation task; these were selected by the authors on the basis that together the samples would generate the largest and most diverse range of perceptual experiences for participants. Remaining samples were introduced across the focus groups.

**Table 2:** Matrix of music samples selected for the individual elicitation task.

Sample	Original	Car noise	Compression	Band-pass	Object-based audio
Charlie	X		X		
Chicken	X		X		
Improv	X	X			
Lush [lyrics]	X	X		X	Vocals −6 Other +6
Perfect Day [lyrics]	X				Other −6 Bass +6
Promise [lyrics]	X	X			Vocals +6 Drums −6
Mendelssohn	X		X	X	
Save Me [lyrics]	X				
Stars [lyrics]	X			X	Bass −6 Drums +6
Verdi	X				
Total	10	3	3	3	8

### 2.3 Data analysis

Quantitative data analysis was performed in R (R Core Team, 2023). Perceptual terms from the individual elicitation stage were manually cleaned (i.e., removing suffixes and adverbs, retaining only single-word terms and base words). Then these data were summarized by means of descriptive statistics, such as frequency distributions of terms across the elicitation task overall and between aided and unaided listening. Focus groups were audio recorded and analyzed by the first and second authors. Data from the follow-up stage were analyzed to explore the perceptual attributes and rating scales developed by participants across the focus groups, in terms of how accurately the attribute definitions reflected discussions, and how easy the rating scales were to use.

## 3 Individual elicitation of perceptual terms

In the first stage, participants completed an online individual elicitation task, listening to 27 music samples twice (once aided, once unaided) and providing up to three single-word terms to describe each sample in terms of audio quality. For aided listening, participants were instructed to use the hearing aid settings or program that they would normally use for music. No direct definition of ‘audio quality’ was provided to participants, to avoid biasing responses and allow participants to navigate the meaning of this concept across the study process; however, some suggestions of what audio quality might refer to were provided at the start of the task (see Supplementary material), to aid understanding. Music samples were administered to participants in a randomized order within the aided and unaided blocks, and participants could listen to each sample as many times as required. Before starting the task, participants were presented with a test music sample, with which they could set their playback levels to something that was audible and comfortable for them (this was done before both aided and unaided blocks). Finally, participants were instructed to listen to the music samples through a set of loudspeakers, and not in-built computer speakers, headphones, or earphones. Notably, this design introduces limitations in control over listening environments but was considered an appropriate balance for ecological validity and diversity of perceptual experience (see Discussion).

### 3.1 Results

In total, 1,438 responses were elicited from participants across music samples, generating 373 unique terms. Of these unique terms, 172 terms were used more than once, 114 terms used more than twice, and 89 terms used more than three times. The 20 most frequent terms across the overall task are presented in Table 3, with ‘clear’ being notably more frequent than other terms.

**Table 3:** Twenty most frequently used terms across the individual elicitation task.

Term	Frequency	Term	Frequency
Clear	111	Tinny	21
Loud	44	Okay	20
Distorted	39	Bright	19
Balanced	38	Harsh	19
Unclear	33	Blurred	18
Indistinct	26	Echoey	18
Noisy	26	Flat	17
Muddy	25	Narrow	16
Muffled	23	Sharp	16
Distant	21	Bassy	14

**Figure 1:**
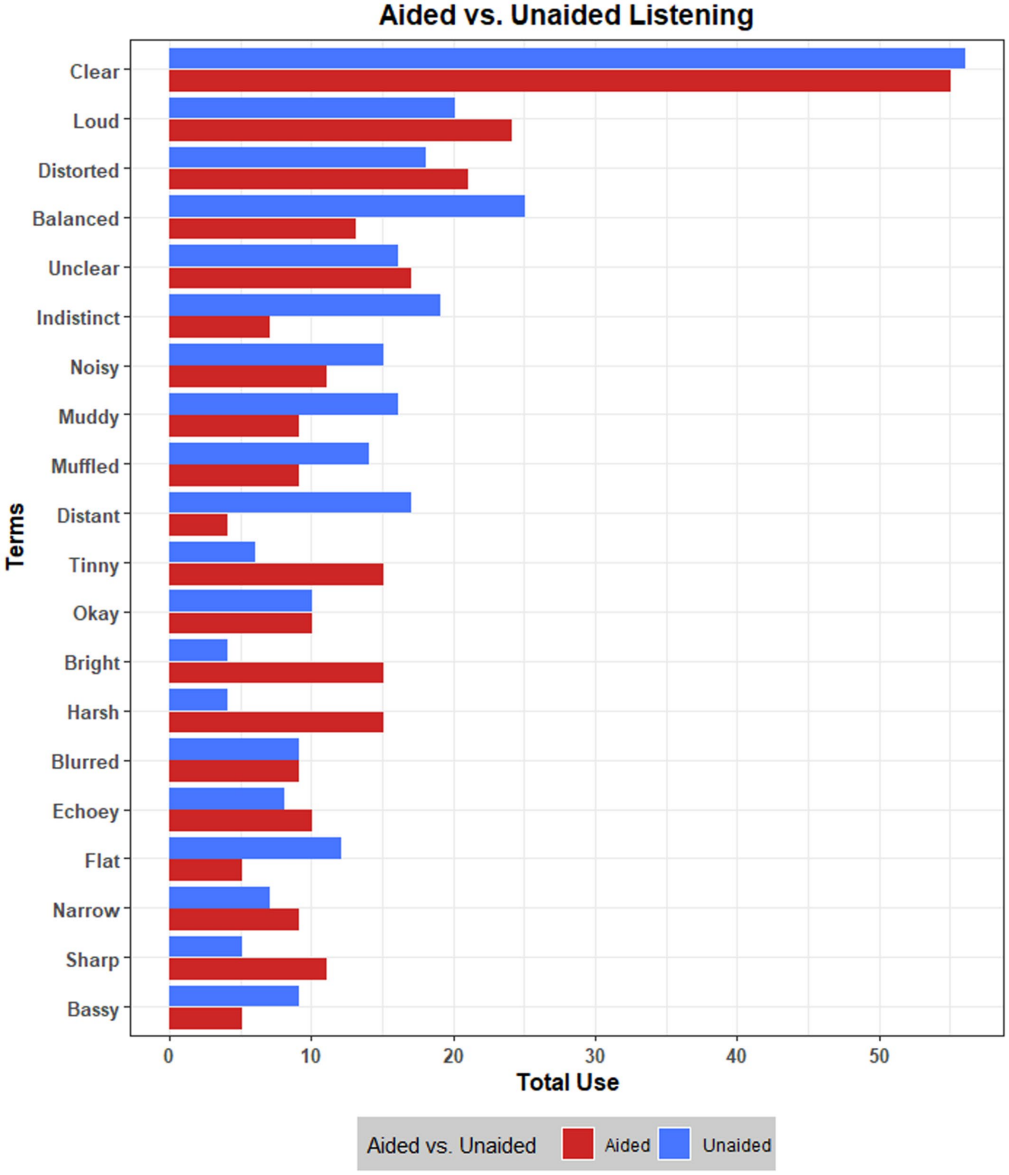
Total use of 20 most frequent terms across aided and unaided listening.

The total use of the top 20 terms was compared across aided and unaided listening, visualized in Figure 1. Although these terms are used similarly by participants across aided and unaided listening, there were some descriptive differences. For example, listening without hearing aids seemed to result in the audio sounding ‘muddy’, ‘muffled’ and ‘distant’, compared to with hearing aids; contrastingly, aided listening appeared to result in the audio being described more often as ‘harsh’, ‘bright’, and ‘tinny’.

Further explorations of data were performed at the levels of processing conditions, individual music samples, and individual participants. Although these results provide little central insight for this study, they are accessible in the Supplementary material.

## 4 Focus group 1

The aim of focus group 1 (FG1) was to familiarise participants with the perceptual term space from the individual elicitation task, discuss what terms may be most important for their music listening experiences, and explore how these terms may relate to each other.

**Figure 2:**
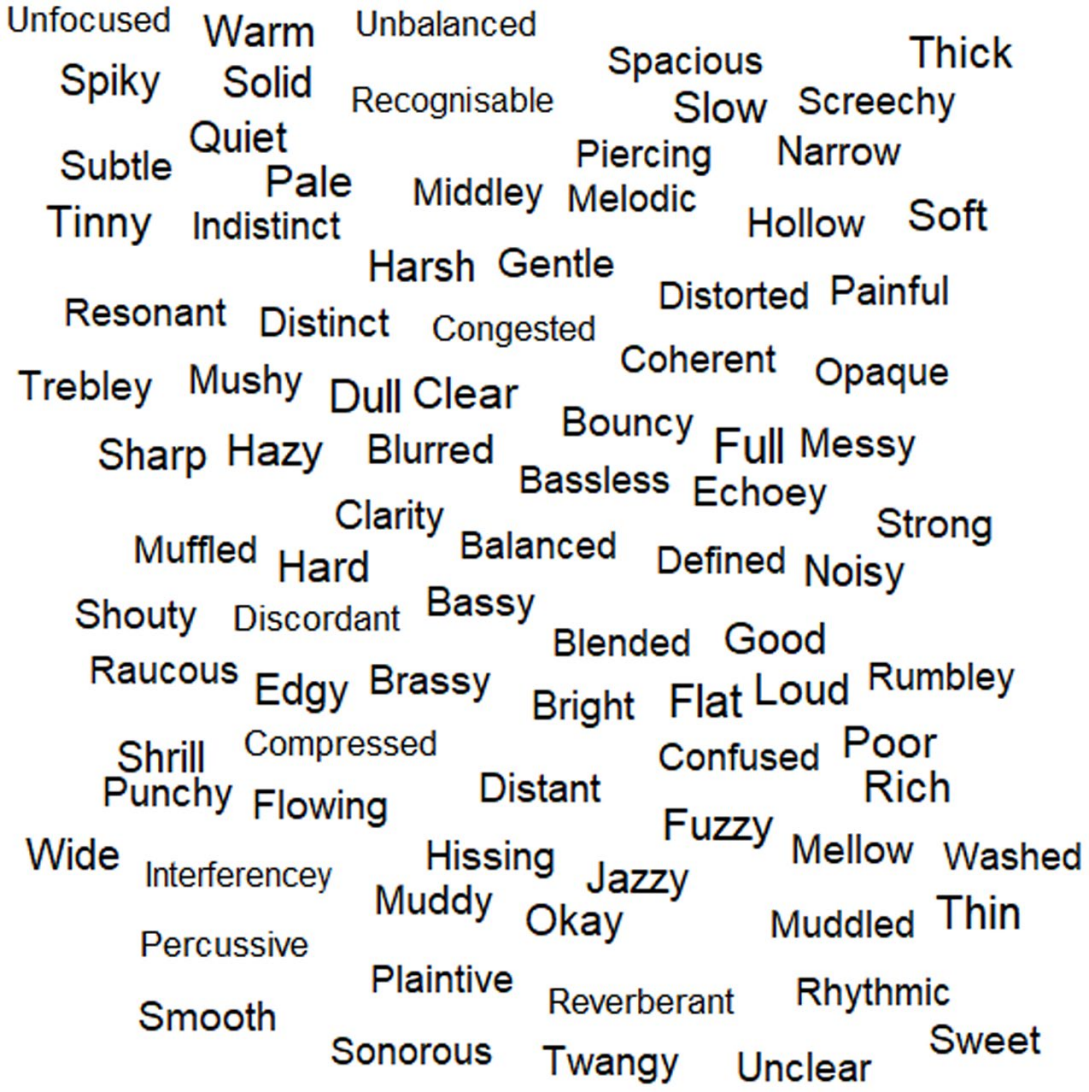
Wordcloud visualization provided to participants in FG1, presenting terms used more than three times in the individual elicitation task; there is no correspondence between word size and frequency of use.

In the morning session, participants were given a word cloud visualization (see Figure 2) of perceptual terms used four or more times across the individual elicitation task (*n* = 89); this was deemed a reasonable and simplified starting point to enable discussion, and it was expected that this space reflected the more important perceptual terms given their frequent usage. The word cloud was produced using the ‘wordcloud’ R package (Fellows, 2018). Participants were then asked to listen to 8 music samples from the individual elicitation task and choose a single perceptual term from the word cloud that best described their experience of each, in relation to audio quality. Samples were played through stereo loudspeakers in the focus group space, and participants wore their hearing aids (this was the case across all focus groups). Participants were able to choose the same term for multiple samples if desired, but the focus was not on how many times a term was chosen. The aim of this task was to further reduce the perceptual term space and capture the most important terms to take forward in discussions. Samples were chosen so that there were two for each of the processing conditions of *original*, *compressed*, *bandpass*, and *car noise* (object-based audio was omitted due to time constraints). The pairs of samples within each processing condition were selected to represent a diverse perceptual term space, driven by the individual elicitation data. To achieve this, Manhattan distance matrices were generated for samples within each processing condition, based on term data (see Supplementary material); this was achieved using the ‘factoextra’ R package (Kassambara and Mundt, 2020). The sample pairs demonstrating the largest distance were selected for inclusion in this focus group task; where this procedure resulted in duplicated samples, the next largest distance was selected within a processing condition, to ensure the inclusion of eight different samples.

In the afternoon session, participants discussed the reduced term space produced in the morning session, exploring similarities or differences between terms, and how they may be possibly grouped together or separated in relation to their meaning. Participants were given no starting point for discussions, to avoid biasing the process.

### 4.1 Results

In the morning session, a total of 44 perceptual terms were selected by participants from the 89 terms in the word cloud, to describe the audio quality of the eight music samples played. These terms are presented in Table 4.

**Table 4:** Terms retained by participants in the process of the morning session of FG1, in no particular order.

Terms				
Percussive^*^	Jazzy^*^	Strong	Bouncy	Punchy
Sonorous	Rhythmic^*^	Spacious	Muddy	Noisy
Blurred	Indistinct	Defined	Discordant	Messy
Flat	Distorted	Mushy	Congested	Muddled
Screechy	Confusing	Narrow	Piercing	Twangy
Sharp	Resonant	Clear	Poor^*^	Tinny
Harsh	Thick	Brassy	Shouty^*^	Thin
Compressed	Hollow	Bassless	Echoey	Interferencey
Rumbley	Muffled	Hazy	Unclear	

^*^These terms were removed by participants in the afternoon session of FG1.

In the afternoon session, these terms were discussed. Participants raised the possibility that not all terms were audio quality descriptors, but instead described the *music* itself; some examples include ‘percussive’, ‘rhythmic’, ‘jazzy’. Participants removed these terms, alongside others deemed either too specific to certain music styles (e.g., ‘shouty’ and vocal music) or as more judgmental rather than descriptive (e.g., ‘poor’).

Participants discussed seemingly related terms including ‘muddled’, ‘blurred’, ‘indistinct’, ‘messy’, ‘mushy’, ‘muddy’, ‘hazy’ and ‘unclear’. They focused on possible differences in meaning across these terms, despite some acknowledged similarity between them; for instance, ‘muddled’ appeared to differ in some ways to ‘muddy’, with one participant linking ‘muddled’ to a confused or ‘jumbled’ combination of instruments and sound sources, and ‘muddy’ to a sense of ‘blurred’ or ‘noisy’ sound. Participants were asked to consider if these terms have antonyms, resulting in further terms including ‘clarity’, ‘distinct’, ‘open’, ‘clear’, ‘transparent’ and ‘focused’.

**Figure 3:**
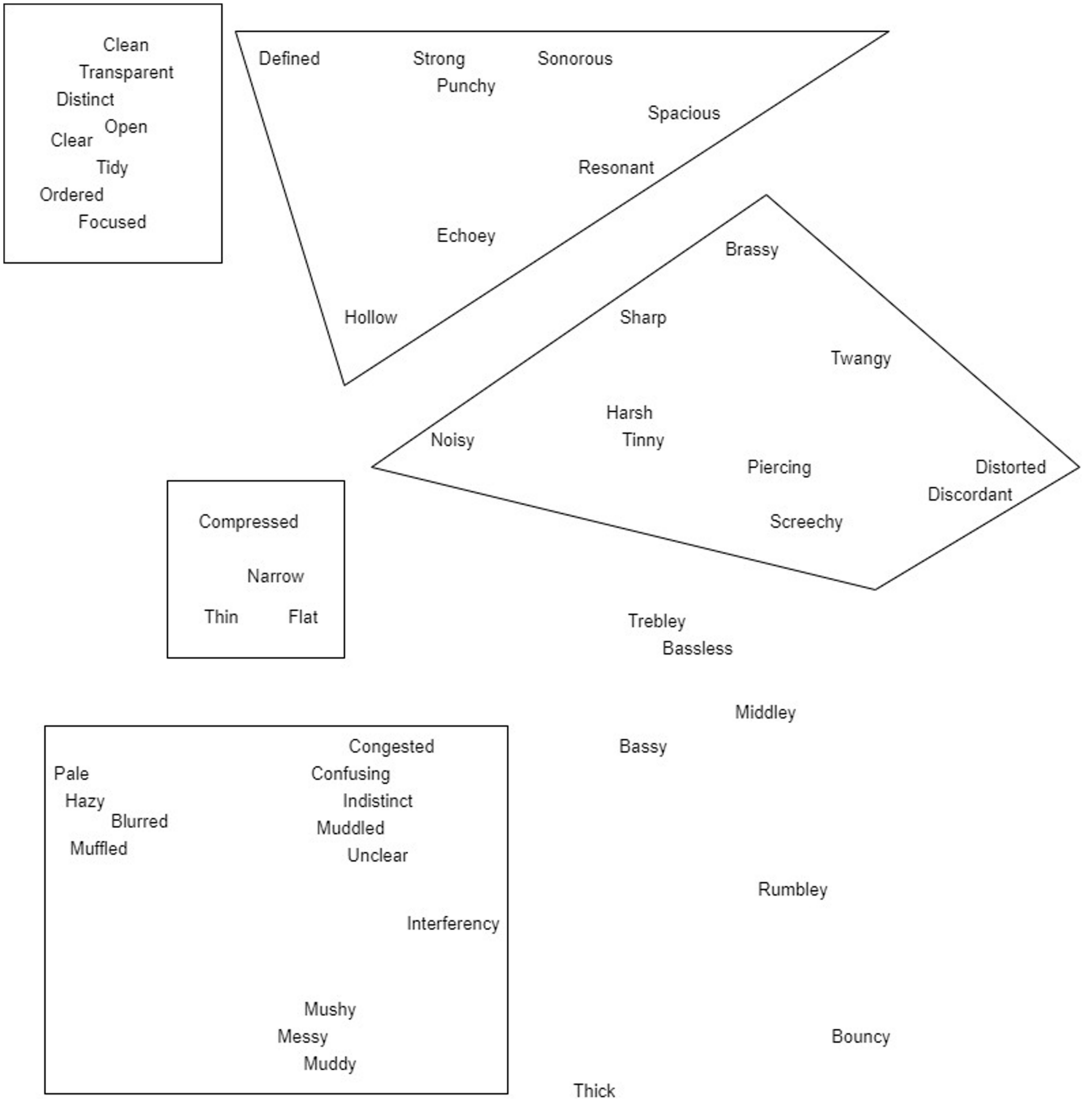
Spatial map of perceptual terms, arranged by participants in FG1. Loose groupings of terms were proposed by the researchers to facilitate discussions in FG2, and these are visualized.

To conclude FG1, participants moved iteratively through the perceptual terms retained from the morning session (Table 4) and were asked to position each term spatially (i.e., on a whiteboard) in relation to their similarity in meaning; terms placed closely together were considered by the group to be similar in meaning. As a result, FG1 concluded with a spatial map of the perceptual terms, presented digitally in Figure 3. It is worth noting that some new terms were raised by participants during the afternoon discussions (e.g., to contextualise meaning), and were included in the spatial map.

## 5 Focus group 2

The aim of focus group 2 (FG2) was to discuss and arrive at finalized groupings of perceptual terms, with these groupings to be taken forward to form perceptual attributes (with labels, definitions, and measurement structures) in the final focus group. In FG2 participants were provided with the spatial map from FG1, and two word clouds: the first was a version of the word cloud from FG1, but with perceptual terms not retained in the morning session of FG1 highlighted in red; the second was a visualization of terms used in the individual elicitation task more than once, but less than four times. These materials were provided for reference, and to encourage them to consider whether there were any important aspects of audio quality that had been omitted through the design of the sensory panel process. To facilitate discussions, the researchers proposed several loose term groupings from the spatial map based on analysis of FG1 discussions; these groupings did not encompass every term between them. For each grouping, two samples from the individual elicitation task were selected, by identifying which samples were described most frequently using terms from each grouping. The loose groupings are indicated in Figure 3.

In the morning session, participants used the spatial map from FG1 as a foundation, first discussing terms that appeared to group more closely together. As the work continued into the afternoon session, participants were tasked with working through progressively less defined term groupings from the spatial map. FG2 concluded with a finalized grouping structure of perceptual terms.

### 5.1 Results

Participants first discussed a close grouping of terms including ‘congested’, ‘confusing’, ‘indistinct’, ‘muddled’, ‘unclear’, ‘messy’, and ‘muffled’ (see Figure 3), and listened to related samples from the individual elicitation task. There was discussion around the difference between ‘muddled’ and ‘muddy’, reflecting FG1; ‘muddy’ was described by some as a lack of clarity, and ‘muddled’ may be a confused combination of sound sources. As an initial refinement, the group agreed that the term ‘confusing’ was not the same as the others in this group and was removed. The term ‘opaque’ was taken from the word cloud as something that had not survived the FG1 process but was considered important for this term grouping. A point was made that ‘clear’ and ‘unclear’ may be opposing ends of a dimension, and there was agreement on the links between the related term groupings here. There were some questions around whether ‘clear’ refers to how well you can hear the music as the composer intended, or the logic of the music itself; however, this led to recurrent discussion of describing music vs. audio quality, with agreement that this is about describing the sound (i.e., the quality of the reproduction), rather than the performance or composition. Finally, terms such as ‘pale’, ‘hazy’, ‘blurred’ and ‘muffled’ were separated from the remaining terms of this loose grouping, though were not taken further as a finalized separate group.

Participants then focused on terms such as ‘clean’, ‘transparent’, ‘distinct’ and others within this loose grouping (see Figure 3). After listening to related music samples, participants suggested that these terms reflect being able to pick out different sounds or elements, and that the sound feels balanced; relatedly, there was some consideration of adding the term ‘balanced’ to the group, but no complete agreement was reached. Participants removed terms such as ‘tidy’ and ‘ordered’, outlining how these terms may feel more musical; this led participants to revisit the first term grouping and remove ‘messy’. Some terms were added to the group, including ‘clarity’ (from the word cloud), and ‘recognisable’ as requested by a participant. The term ‘recognisable’ was discussed at length, negotiating whether this meant recognising a familiar piece, or recognising qualities of the sound; the group agreed this was not about memory or familiarity, but about recognising elements related to audio quality.

Participants then moved to terms such as ‘brassy’, ‘sharp’, ‘discordant’, ‘harsh’ and others (see Figure 3), with related samples played to them. Some participants highlighted ‘piercing’ as important, and others noted the painful, discomforting, or overwhelming experience of listening to the samples. Excess treble frequencies were proposed as linking terms such as ‘harsh’, ‘piercing’, ‘tinny’ and ‘sharp’. Interestingly, some participants did not like terms such as ‘sharp’ and ‘flat’ due to their musical connotations (e.g., sharp or flat notes or pitch), with some suggestions that ‘shrill’ could be a better term (taken from the word cloud). Discussions transitioned to a focus on terms such as ‘trebley’, ‘middley’ and ‘bassy’. Notably, ‘trebley’ was felt to be quite dissociable from ‘harsh’, ‘piercing’ and similar terms; however, these frequency-related terms were kept together as a grouping, with participants agreeing that frequency or pitch balance is important for their experience. Participants found ‘tinny’ difficult to connect with other terms; similarly, ‘distortion’ appeared to be somewhat separable from terms outlined above, with some participants linking this to ‘noisy’ or ‘fuzzy’ (a term used that was not present in the spatial map). One participant suggested the term ‘bright’ (taken from the word cloud), but there was some consensus that this does not capture the more painful side of treble qualities in sound. Furthermore, ‘twangy’ and ‘brassy’ were dropped, either for being too specific to certain musical styles or instrumentation, or as describing musical qualities more than audio quality.

As a product of this discussion, ‘distortion’, ‘noisy’ and ‘tinny’ were grouped together. When targeting this, participants found agreement in describing ‘distortion’ as something in the sound (e.g., artefacts) that should not be there, or that was not there in the original production. Overall, the group felt that this term belonged on its own and was important for their experience; ‘noisy’ was dropped, although ‘tinny’ was retained loosely in relation to terms such as ‘harsh’, ‘piercing’ and others.

When discussing the term grouping including ‘compressed’, ‘flat’, ‘narrow’ and ‘thin’, various perspectives were put forward. Some participants referred to the dynamic range of the sound, others referred to the frequency or pitch range, and occasionally some of these terms were used to talk about spaciousness in the sound quality. Some participants felt that ‘compressed’ and ‘flat’ were distinct from ‘thin’ and ‘narrow’. Overall, however, ‘compressed’ was judged to be quite technical and different in relation to the other terms and was removed by the group. There was no consensus or certainty on this term grouping, but it was retained at this stage as some participants felt it could be important.

Finally, the group navigated the remaining broad grouping from the spatial map, including terms such as ‘echoey’, ‘resonant’, and ‘spacious’ amongst others. Participants struggled with navigating this grouping, but the discussion gravitated towards aspects of space in relation to sound quality. After playing some samples linked to related terms in the individual elicitation task, a participant described the sound as ‘echoey’ but not ‘spacious’; in their elaboration, it was suggested that the sample sounded confined, with little space. Another participant felt that ‘spacious’ described a sense of being in a space, as opposed to characterising audio quality. Some additional terms were highlighted, including ‘distant’ and ‘reverberant’ (from the word cloud). Overall, the group agreed that the term grouping could be narrowed down to a focus on space, and this was retained for further discussion as a potentially important perceptual attribute.

FG2 concluded with seven perceptual term groupings, outlined as follows:

1. Clear, clean, transparent, distinct, open, focused, clarity, recognisable.
2. Congested, indistinct, muddled, unclear, opaque, mushy, messy, muddy.
3. Trebley, middley, bassy, bassless, balanced.
4. Piercing, harsh, screechy, shrill, sharp, tinny.
5. Wide, narrow.
6. Distorted.
7. Spacious, resonant, echoey, distant, reverberant.

Importantly, during FG2 participants discussed the potential relevance of ‘loudness’ to their perceptual experience of music audio quality, given that this term was one of the most used in the individual elicitation task (Table 3). However, participants broadly agreed that the term was perhaps confusing, or did not effectively explain their experiences. Additionally, one participant noted that whilst ‘loudness’ may have been used often in the individual elicitation task, the group had since gained knowledge regarding their perceptual experiences of music, possibly resulting in ‘loudness’ no longer feeling as important.

## 6 Focus group 3

The aim of the final focus group (FG3) was to develop labels and approximate definitions that capture the perceptual attribute encapsulated by term groupings from FG2. A further aim was to generate measurement scales for the perceptual attributes; this involved determining the type of scale, scale endpoints, and scale midpoints if applicable. For FG3 participants were given the word cloud from FG2, visualising perceptual terms used four or more times in the elicitation task and highlighting in red those that were not retained in FG1. In addition, as the development of definitions was anticipated to be difficult in some cases, definitions for similar perceptual attributes in existing literature were prepared (Gabrielsson, 1979; Toole, 1985; Zacharov and Koivuniemi, 2001; Berg and Rumsey, 2003; Choisel and Wickelmaier, 2007; Souza et al., 2007; Lokki et al., 2011; Uys et al., 2012; Lindau, 2015; Pedersen and Zacharov, 2015; Legarth et al., 2016), in case discussions required support. Finally, across all focus group discussions the research team reviewed the data, discussions, and existing literature (Jekosch, 2004; Lorho, 2010; Brunnström et al., 2013; Möller and Raake, 2014; Pike, 2019), to develop a working definition of *music audio quality* and rating scale structure; this was proposed in FG3 for feedback from participants.

In the morning session, participants discussed the first three perceptual term groupings in the list above, working towards labels for the overall perceptual attribute, rating scale structures, and rough definitions. In the afternoon session, this process was continued for the remaining perceptual term groupings. To conclude FG3, participants were asked if there were any important perceptual experiences that had been overlooked, and were presented with a working definition of *overall audio quality* for feedback.

Although all 12 participants attended FG1 and FG2, 10 attended FG3 due to university strike action in February 2023.

### 6.1 Results

Discussions began with the perceptual grouping of ‘clear’, ‘clean’, ‘transparent’ and others. Participants agreed that this group, and the group containing ‘congested’, ‘indistinct’, ‘muddled’ and others, were two ends of the same dimension. Consequently, participants considered this to be a single perceptual attribute labelled *clarity*, with ‘clear’ and ‘unclear’ as scale endpoints. By *clarity*, participants referred to the ability to hear different pitches and rhythms, and to detect and separate the various sound sources in the music, without necessarily requiring explicit knowledge of what the sound sources are. Some focus was placed on the term ‘recognisable’, but the group agreed that ratings of *clarity* should *not* be based on explicit memory or familiarity with a piece of music, or in reference to how music may have been heard before the onset of hearing loss; indeed, the group highlighted those cases in which hearing loss is not acquired but is congenital. Concurrently however, participants acknowledged the importance of expectations more broadly, with each listener having a sense of how music ‘should’ sound within different stylistic frames.

Participants then discussed the grouping of ‘trebley’, ‘middley’, and ‘bassy’. Participants understood that this grouping captured aspects of frequency balance, or balance in the reproduction of the music. During this discussion, participants appeared to talk naturally in terms of ‘too much’ treble or bass, and as such were conceptualizing this perceptual attribute in terms of what they would prefer as individuals; however, when challenged on this, participants agreed that this felt more like preference judgment, rather than describing perceived audio quality. Thus, instead of ‘too much treble’ (for example), participants considered describing this as ‘very trebley’. There were suggestions that treble could encapsulate terms such as ‘tinny’, ‘harsh’ and ‘shrill’, although no consensus was reached with this. Participants were asked to consider how many scales would be needed to sufficiently capture this frequency-related perceptual attribute. When participants discussed using three scales, these reflected *treble strength*, *middle strength*, and *bass strength*. When discussing a two-scale approach, the *middle strength* scale was dropped, reflecting a consensus on middle frequencies being less important. With a single scale, participants tended towards an agreement on relative *frequency balance* between treble and bass being captured, with a balance between the two reflecting a midpoint on the scale. The participants were asked to vote on the preferred approach, with three scales being preferred by a small majority (i.e., five out of nine; one participant did not vote). It is worth noting that some participants had difficulty accessing discussions of frequency balance; to support this, music samples were played at their request to reflect discussions, and when contextualised in terms of pitch (a perceptual construct closely linked to frequency), these participants were able to relate to the perceptual attribute more directly. Participants also chose to listen to these music samples with and without their hearing aids to exemplify how the frequency balance can change. Removing hearing aids resulted in perceptions of the music being ‘less trebley’ for most, which may reflect increased gain applied within hearing aids to compensate for loss in high frequencies.

The sensory panel continued to discuss the next term group, encompassing ‘piercing’, ‘harsh’ and others. This was consistently considered a negative property of perceived audio quality, described as painful, ugly, overwhelming, and overamplified. In FG2, grouping of these terms appeared to tap into a painful experience of treble frequencies or higher pitches; interestingly however, in FG3 participants discussed whether these terms should be applicable across the frequency spectrum. For instance, one participant noted how they could imagine a bass sound to be ‘piercing’, but not ‘shrill’. Whilst this was an attractive idea to participants, it was difficult to reach a consensus, and they returned to a focus on treble sounds, after noting that there was not a natural collection of terms that mirrored this perceptual group for bass sounds in the word cloud resources provided. To confirm this, the researchers communicated a definition of *sharpness* from the literature that subverted treble-specific aspects, and this was not considered by participants to reflect the meaning of their perceptual grouping. Overall, the perceptual attribute was labelled as *harshness*, with scale endpoints being ‘not harsh’ to ‘very harsh’.

Participants then discussed ‘distortion’. Following FG2, it was suggested that distortion may refer to something in the perceived audio quality that should not be there; this may include hisses, pops, or crackles, and distortions of pitch, especially from the perspective of listeners who may have diplacusis. Participants highlighted related terms or concepts, such as accuracy, authenticity, or the sound as being representative of the composer’s intention. These concepts were discussed, and participants navigated ambiguities, such as whether distortion would then refer to performer error. One participant suggested that distortion may reflect artefacts that were not produced by the original motor production of sounds, or through their purposeful recording and reproduction. An interesting example was raised by a participant, asking whether music played by a child with no instrumental or musical education on a piano (i.e., bashing keys indiscriminately) would sound distorted; however, another participant claimed that distortion would only be present if what is perceived is not true to the sounds that were produced. An alternative example raised by another participant was that if a performer played a note at 440 Hz, but they perceived the note as at 452 Hz, then this is distortion; however, if the performer accidentally played the 440 Hz note at 452 Hz, then this is not distortion. In concluding these discussions, participants labelled the perceptual attribute as *distortion*, with scale endpoints of ‘not distorted’ and ‘very distorted’.

The final participant discussions focused on the term groups involving ‘spacious’, ‘resonant’ and ‘echoey’, and ‘wide’ and ‘narrow’. Firstly, following uncertainties from FG2, participants reflected on the terms ‘wide’ and ‘narrow’, and a consensus was quickly reached, outlining these terms as unintuitive, less meaningful, and as referring to too many possible aspects of audio quality; consequently, these perceptual terms were dropped by the participants. In terms of the other perceptual group, there was again some disagreement around the similarity of meaning across terms such as ‘spacious’, ‘resonant’ and ‘echoey’, reflecting FG2. Some participants further felt that a term like ‘spacious’ was quite technical or musical in its meaning. One participant asked whether the perceptual attribute being captured here referred to quality of the room or performance space, referring to ‘dead’ spaces, where sound is absorbed, as reflecting one end of a possible scale. However, there was difficulty in navigating this grouping. As such, the researchers communicated a few related definitions from existing literature, that reflected how sounds are located in the space or sound image, how well the space of the music recording is perceived, or the effects of reverberation. Participants were asked whether ‘spacious’ may be referring to some effect of room colouration on the sound quality. One participant noted that whilst they understand this concept, they would never use it to describe audio quality. For some participants with music performance experience, it was clear that ‘spacious’ and related terms were perhaps more important, with numerous anecdotes shared regarding how the sound of a performance is affected by the space in which it takes place. However, some participants perhaps felt that this concept was less intuitive for music recordings. Whilst no definitive consensus was reached for this perceptual grouping, participants labelled this as *spaciousness*, with some suggestion that the scale endpoints could be ‘no echo or reverberation’ to ‘lots of echo or reverberation’.

In concluding FG3, participants were provided a working definition of *overall audio quality*, with scale endpoints being ‘very poor’ and ‘very good’. Qualitatively, feedback was highly positive, with participants agreeing that the definition reflected the meaning of audio quality that was navigated and constructed as a group. However, to determine further how well definitions and rating scale structures reflect the meaning and consensus of the sensory panel participants, an online follow-up task was performed.

## 7 Follow-up task

In FG3, participants agreed on seven perceptual attributes, and developed labels, scale endpoints, and approximate consensual definitions. Discussions were analysed, leading to researcher-led proposals of rating scale structures and working definitions of perceptual attributes to reflect the meaning agreed by the participants. The attributes, definitions and scale structures are presented in Table 5.

**Table 5:** Perceptual attributes developed across the focus groups, alongside their working definitions, and scale structures (all scales use a continuous range of 0–100).

Attribute	Definition	Scale endpoints
Clarity	Clarity refers to how well you can hear the different elements of the music, including being able to distinguish between the different sound sources, instruments, or voices in the music, and being able to hear the qualities that distinguish one sound source from another. Unclear music may sound indistinct, mushy, or muddy; clear music may sound clean, distinct, and transparent.	Very unclear – Very clear
Harshness	Harshness refers to an emphasis or amplification of certain sound qualities (often in the treble frequencies or higher pitches) that can feel overwhelming, abrasive, painful, or discomforting. Harsh sounds may sound piercing, screechy, shrill or sharp.	Not harsh – Very harsh
Distortion	Distortion refers to a sense that the audio quality of the music contains elements that should not be there, that do not feel right, or that have appeared between the music’s reproduction and your listening of it. These elements may include artefacts (e.g., noise, hiss, pops, crackles), or distortions to pitch (e.g., the pitches sound wrong compared to what you imagine was performed and recorded). No distortion may reflect a sense that the music is an authentic or accurate representation of what was performed and recorded, with no sense of pollution, interference, or distortion in the audio signal.	Not distorted – Very distorted
Spaciousness	Imagine hitting a drum in two spaces: a small living room, and then a large cathedral. Whilst your action is the same, the sound produced can take less time or more time to return to silence, as it reverberates in a space. Spaciousness refers to the perceived presence of these reverberations created by the space in which the music was performed. This may refer to how much you feel the music is ‘coloured’ by this space. A lack of spaciousness may mean that reverberations or a sense of space is not heard, with the opposite true for very spacious sound.	Not spacious – Very spacious
Treble strength	Treble strength refers to the perceived strength or prominence of sound qualities that are characterised by higher frequencies in the treble range, or similarly, sounds, instruments or voices with higher pitches.	Not trebley – Very trebley
Middle strength	Middle strength refers to the perceived strength or prominence of sound qualities that are characterised by middle frequencies found between bass and treble ranges, or similarly, sounds, instruments or voices with pitches perceived as being between lower and higher pitches.	Not middley – Very middley
Bass strength	Bass strength refers to the perceived strength or prominence of sound qualities that are characterised by lower frequencies in the bass range, or similarly, sounds, instruments or voices with lower pitches.	Not bassy – very bassy
Frequency balance^*^	Frequency balance refers to the perceived, relative balance between treble (or higher pitches of sound) and bass (or lower pitches of sound) in the audio. Audio described as more bassy would be characterised as having stronger or more prominent bass frequencies and pitches in comparison to treble frequencies and pitches, with the opposite true for audio described as more trebley. The middle point of this scale indicates a perceived balance between bass and treble.	Very bassy – Very treble
Overall audio quality	Perceived audio quality results from judgments of the sound of the music, in relation to a person’s expectations of how the music should ideally sound to them. Imagine listening to a piece of music in two different ways: listening through a cheap mobile phone, and then listening through high quality loudspeakers. The music is fundamentally same in both cases, but the audio quality is very different.	Very poor – Very good

^*^This attribute was proposed as a single-scale alternative to treble strength, middle strength, and bass strength.

To determine how well these definitions aligned with participant discussions, and how usable the rating scales were, an online follow-up task was performed. Participants from the sensory panel (*N* = 10) were first presented with working definitions for each of the seven perceptual attributes (*clarity, harshness, distortion, spaciousness, treble strength, middle strength,* and *bass strength*), and two additional attributes of *overall audio quality* and *frequency balance*; the *frequency balance* attribute was proposed as a reflection of discussions around balance between bass and treble in FG3, and as a more concise alternative for future empirical work where fewer rating scales may be more practical. Alongside each definition, a shorter concise definition was proposed (see Supplementary material) to improve readability. Participants were then asked to use the attribute rating scales (each a continuous scale with a range of 1 to 100) to score five music samples from the individual elicitation task. Sample presentation order was randomised, as was the ordering of attribute rating scales (except for *overall audio quality,* which was always the final scale). Sample selection was motivated by individual elicitation data, and on the basis that the five samples would afford use of the full range of the rating scales. For each rating scale provided. After listening, participants were asked to rate how accurately the working definitions of each perceptual attribute reflected their understanding of the consensus meaning from the focus groups (continuous scale, 1–100), whether the shorter alternative definitions maintained this meaning (yes/no), and how easy it was to use the scales to score the five samples (continuous scale, 1–100).

### 7.1 Results

**Figure 4:**
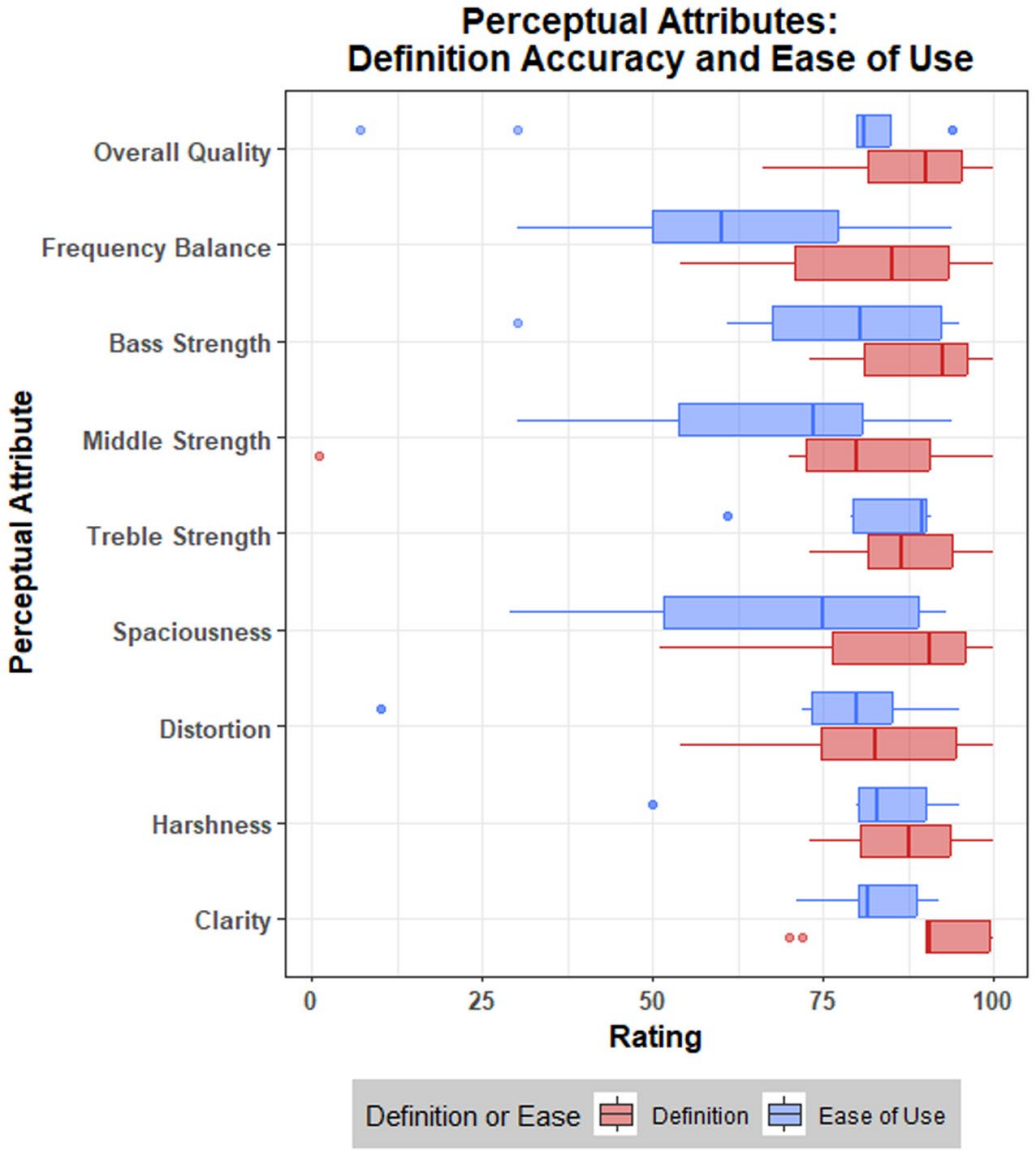
Boxplot visualization of ratings, across nine attributes, for accuracy of definition and ease of use for the attribute scales.

Data for the accuracy of attribute definitions and ease of use for the attribute scales are presented in Figure 4 and Table 6.

**Table 6:** Mean (SD) ratings for accuracy and ease of use ratings.

Attribute	Accuracy of definition	Ease of use
Clarity	90.10 (10.99)	82.80 (6.86)
Harshness	87.40 (8.92)	79.30 (16.20)
Distortion	82.50 (14.71)	68.20 (31.35)
Spaciousness	82.80 (18.25)	67.50 (18.25)
Treble strength	87.70 (8.95)	82.20 (12.00)
Middle strength	75.30 (27.98)	66.90 (21.56)
Bass strength	88.80 (9.24)	76.50 (20.29)
Frequency balance	81.10 (16.29)	61.88 (23.66)
Overall audio quality	86.90 (11.76)	71.60 (28.97)

Data suggest that the working definitions provided to participants for each perceptual attribute accurately reflected the consensus meaning from the focus groups. Across all attributes, the mean rating of ‘accuracy’ was 84.73 (SD = 15.24). A single rating of 1 was provided by a participant for *middle strength*, although this appeared to be an outlier (see Figure 4), given that the range of ratings across attributes once this value was removed was 51–100. The lowest mean rating for accuracy was attributed to *middle strength* (M = 75.30 M = 83.55 with outlier removed; see Table 6).

Ease of use ratings were generally lower, but still reflect positive responses, with a mean rating of 73.11 (SD = 22.04) across attributes. There were three notably low ratings (see Figure 4), such as a rating of 7 for *overall quality* and two ratings of 10 for *distortion.* One participant provided feedback for their distortion rating, stating: “*It was difficult because it conflates distortion with dissonance* (I.e., *‘pitch sounds wrong’*).” The lowest mean ratings for ease of use (see Table 6) were attributed to *frequency balance* (M = 61.88), *middle strength* (M = 66.90), *spaciousness* (M = 67.50), and *distortion* (M = 68.20). These results reflect focus group discussions and lesser consensus or importance for these attributes.

Finally, participants strongly agreed that the shorter attribute definitions retained the meaning of the full definitions, with no negative responses given. Beyond these results, whilst there was no immediate interest in additional levels of interpretation in the data given a relatively small sample size, see the Supplementary material for visualizations of attribute ratings across the five music samples.

## 8 Discussion

Whilst work has explored audio quality perception in ‘normal’ hearing listeners, research with hearing-impaired listeners is scarce. This study worked with a sensory panel of hearing aid users to take initial steps towards understanding the important perceptual attributes and characteristics of music audio quality. Adopting sensory evaluation methods, a series of tasks and focus groups were completed, with the sensory panel discussing and reaching a consensus on seven important perceptual attributes of audio quality: *clarity, harshness, distortion, spaciousness, treble strength, middle strength,* and *bass strength*. Below, we summarise and contextualise these attributes in relation to existing research, reflect on study limitations, and outline implications and future directions.

### 8.1 Perceptual attributes of music audio quality

#### 8.1.1 Clarity

*Clarity* referred to the capacity to distinguish between different instruments, voices, or sound sources in the music, and to hear the qualities that separate sounds from each other; importantly, this perception would not require explicit knowledge of what the sound sources are. This attribute was perhaps most important for the participants, reflecting recent work which has shown that hearing aid users can experience difficulty with hearing out melodies, identifying instruments and hearing lyrics (Madsen and Moore, 2014; Greasley et al., 2019; Greasley, 2022) and often describe music as sounding indistinct, muffled, and lacking fidelity (Greasley et al., 2020b). Similar dimensions are proposed in existing audio quality literature. For instance, *clarity* resembles a dimension of the same label developed by Gabrielsson (1979), Gabrielsson and Sjogren (1979), and Gabrielsson et al. (1988), and another dimension with the same label from Toole (1985) and Lindau (2015). Other comparable attributes include *clear* (Choisel and Wickelmaier, 2007), *definition* (Lokki et al., 2011), *detailed* (Pedersen and Zacharov, 2015), and *source separation* (Legarth et al., 2016). These attributes encapsulate a dimension of audio quality that reflects the distinguishability and separability of sound elements or sources, and the current sensory panel highlighted the importance of this quality in the context of music listening and hearing loss. It is worth noting that the terms used by participants to develop *clarity* resemble some adjacent attributes in the literature, such as *clean* (Pedersen and Zacharov, 2015) and *crispness* (Uys et al., 2012); these attributes refer to terms like ‘dull’, ‘blurred’, ‘distinct’ and ‘muddy’, but do not specifically highlight the experience of distinguishing between sound elements in the audio in their definitions.

#### 8.1.2 Harshness

The next attribute of *harshness* reflected a perceived emphasis of certain sound qualities that can feel overwhelming, painful, or discomforting. Participants agreed that this mostly occurs with sounds in the higher frequencies, though noted that it might also happen in lower frequency sounds. This reflects recent findings which show that sounds are often experienced by hearing aid users as ‘harsh’, particularly in the high frequencies (Greasley, 2022). *Harshness* may be comparable to some perceptual dimensions proposed in previous research, such as *softness* (Gabrielsson, 1979), and *sharpness* (Souza et al., 2007); however, these dimensions make no reference to frequency characteristics in their definitions, instead positioning descriptions of ‘softness’ and ‘gentleness’ as opposing ‘shrillness’, ‘hardness’ and ‘sharpness’. Lindau (2015) also listed *sharpness* as a perceptual attribute in the context of spatial audio, noting that a ‘sharp’ sound may come from emphasised higher frequencies. Sharpness as a concept may resonate with *harshness*, although participants felt that it contained unhelpful musical connotations (e.g., sharp pitches). Interestingly, when discussing harshness, several participants mentioned that listening without hearing aids can ameliorate this perceptual experience and suggested that perhaps their hearing aids go too far in amplifying higher frequencies. Although data from the individual elicitation task were summarised descriptively, results suggest that music samples were described more often as ‘harsh’, ‘bright’ and ‘tinny’ when listening with hearing aids compared to without (see Figure 1).

#### 8.1.3 Distortion

One of the main challenges with music reported by hearing aid users is the experience of distortion (Madsen and Moore, 2014; Fulford et al., 2015; Greasley et al., 2020b), including pitch distortion due to reduced frequency selectivity of the cochlea (*cf.* Moore, 2016), and distortion resulting from limitations of hearing aids for processing music signals (e.g., artefacts). Given these findings, it is not surprising that this was identified as an important sound quality attribute in the current study. For current participants, *distortion* was characterised as perceiving elements in the sound that a listener feels should not be there, such as artefacts (e.g., noise, pops, crackles, hiss) or pitch distortions. An important aspect to this attribute is that it is sufficient for a listener to have a ‘sense’ or ‘feeling’ that a quality of the sound should not be there, regardless of any objective measures of distortion in the signal. Interestingly, there are some similar perceptual dimensions that reflect this characterisation of *distortion* in previous work: Pedersen and Zacharov (2015) highlight *natural* sounds as being similar to a listener’s expectation without any colouration or distortion; similarly, *naturalness* (Lindau, 2015) is described as having an impression that a sound is aligned with expectations or former experience of the sound; finally, Gabrielsson and Sjogren (1979) described *disturbing sounds* encapsulating noise, hissing and crackling, and suggested that these may be more apparent for people listening through hearing aids. Thus, the perception of *distortion* is affected by previous and current subjective experiences of the listener, such that judging sounds based on this attribute is dependent on a person’s expectations and sense, ‘rightly’, ‘wrongly’ or otherwise, of how an audio signal should sound at source. This characterisation is potentially useful for navigating the myriad interpretations that can be assigned to distortion, ranging from signal artefacts to aesthetic textures in some styles of music (e.g., rock).

#### 8.1.4 Treble, middle, and bass strength

Several attributes encompass the frequency characteristics of audio. For instance, *treble*, *middle* and *bass strength* each reflect the perceived prominence of sounds, and potentially pitches, that would occupy treble, middle and bass ranges, respectively. This division reflects a similar pattern in Pedersen and Zacharov’s (2015) sound wheel. However, in the focus groups participants also considered how frequency characteristics might be captured with one or two attributes, which produced some further insights. For instance, participants reached a level of consensus on removing *middle strength* if only two attributes could be used; this aligns with descriptive data from the follow-up task, with *middle strength* perhaps being less easy to use than *treble strength* and *bass strength*. Furthermore, in prior work, hearing aid users have reporting experiencing too much treble and too much bass, but rarely report experiencing an excess of middle frequencies (Greasley et al., 2020b). Additionally, if using only one attribute, participants appeared also to prioritise treble and bass, with one possible formulation capturing the relative balance between these two frequency ranges in the audio. This resembles some audio quality attributes utilised in extant literature, such as *brightness* (Gabrielsson, 1979; Toole, 1985; Choisel and Wickelmaier, 2007), *tone colour* (Zacharov and Koivuniemi, 2001), *dark-bright* (Lindau, 2015; Pedersen and Zacharov, 2015), and *timbre balance* (Legarth et al., 2016). Consequently, although participants marginally preferred the use of three frequency-related attributes to capture their perceptions of audio quality, a single *frequency balance* attribute was also proposed to them during the follow-up task. This single attribute appeared to be less easy to use, but participant feedback was somewhat mixed: one participant noted that separate attributes for treble, middle and bass were not required, whereas another participant highlighted that *frequency balance* is ineffective when a recording is significantly ‘middley’ in quality.

#### 8.1.5 Spaciousness

The remaining attribute of *spaciousness* is perhaps less prominent or important across the perceptual experiences of these sensory panel participants. This is reflected in some ambiguities or difficulties in reaching a consensus on the meaning of *spaciousness*, and in quantitative descriptive data suggesting that the rating scale for this attribute is more difficult to use than some others. In the current context, *spaciousness* appeared to encapsulate the perception of reverberations in the audio, or the degree of room colouration heard in the sound. The effect of room or space was intuitive for several participants during the focus groups, especially those with some music performance experience. However, not all participants felt strongly that *spaciousness* was an intuitive attribute, even if they understood what it referred to. Indeed, *spaciousness* as a single label may imply numerous dimensionalities of ‘space’ that are touched on elsewhere in audio quality literature. For instance, space may be considered in terms of spatial separation, definition, or positioning of sound sources (Toole, 1985; Zacharov and Koivuniemi, 2001; Berg and Rumsey, 2003), which may be prominent in clear stereo or surround sound images. Perceptions of space might also refer to how well defined or perceived the performance space is, from the qualities of the sound reproduction (Choisel and Wickelmaier, 2007). However, the current attribute appeared to align more closely with effects of reverberation, reflecting similar attributes presented by Lokki et al. (2011), Uys et al. (2012), Lindau (2015), and Legarth et al. (2016). In contrast to *clarity*, *harshness* and *distortion*, it is perhaps less clear as to how important perceptions of *spaciousness* are for hearing-impaired music listeners, and whether the strength of perceived spaciousness depends on the musical engagement (e.g., listening to music recordings at home, or attending live concerts); additionally, perceptions of *spaciousness* may vary in character, quality, and focus, depending on how the music is listened to (e.g., through headphones, earphones, loudspeakers, or other technologies).

#### 8.1.6 Note on ‘loudness’

It is intriguing to note that ‘loudness’ was not adopted by sensory panel participants in this study, as several members of the group suggested that it was not useful in explaining their perceptual experiences of audio quality. Notably, ‘loudness’ was the second most used term in the individual elicitation task, although when asked about this during the focus groups, it was not retained as a key perceptual attribute, potentially due to participants learning more about their experiences as the sensory panel process developed, leading to changed perspectives. Loudness is often presented as an attribute of audio quality in existing research and varied contexts (Gabrielsson and Sjogren, 1979; Lokki et al., 2011 Lindau, 2015; Pedersen and Zacharov, 2015; Legarth et al., 2016; Wilson and Fazenda, 2016). Additionally, recent work highlights that hearing aid users often encounter difficulties with music sounding too loud (Greasley, 2022). Thus, it is perhaps unexpected that loudness was not considered an important perceptual attribute in the current study. One possible interpretation of this could be the complex relationships between different perceptual attributes, particularly the potential for loudness as an underlying driver of *harshness* and *distortion*. *Harshness* is presently characterised by (mostly) higher-frequency sounds that are overamplified and cross some threshold, resulting in pain, discomfort or feeling overwhelmed. Such descriptions can also be found when hearing aid users describe excessive loudness (Greasley et al., 2022b); furthermore, it is intriguing that in work by Gabrielsson and Sjogren (1979) ‘loudness’ was seen to load onto a *sharpness* factor alongside ‘hard’, ‘shrill’, ‘sharp’ and ‘screaming’. In terms of *distortion*, it is understood that distortions in audio signals can be a result of excessive loudness, either through exceeding capacities of sound reproduction technology, or as resulting from signal processing such as compression. Again, hearing aid users report issues with music that is too loud, such that this can often result in experiences of perceived distortion (Greasley et al., 2022b). Although conjecture, it is possible that ‘loudness’ was not considered as an important perceptual attribute by sensory participants, in part due to its correlation or association with both *harshness* and *distortion*, such that ‘loudness’ as an independent attribute provided no further explanatory coverage for their perceptual experiences of music audio quality.

### 8.2 Implications and future directions

The current study has several implications. Principally, this work demonstrates a crucial initial step towards better understanding the perceptual experiences of music listeners with a hearing loss, and those who use hearing aids. Although developments of hearing aid and assistive technologies have long focused on speech intelligibility and quality, it is known that listening to music presents a distinct set of issues to be addressed (Chasin and Russo, 2004; Moore, 2016) and that listeners with hearing loss encounter unique problems with music (Greasley, 2022). To improve hearing aid technologies and other consumer devices to address these difficulties, it is important to better comprehend the perceptual experiences of music listeners with a hearing loss. Much research has been carried out, in varying contexts, on perceptions of audio quality and underlying perceptual attributes (Pike, 2019); however, hearing loss is rarely considered.

A key implication from this work is that whilst the perceptual attributes of music audio quality developed by participants often reflect various attributes proposed in audio quality research more broadly, there are some potentially important distinctions that may reflect experiences of those with varying hearing loss profiles. As mentioned above, ‘loudness’ is a prevalent attribute in existing research which did not emerge in the current work. Similarly, in previous research there are attributes related to spatiality and localization of sources in a sound image (Lindau, 2015; Pedersen and Zacharov, 2015), which were not central to the focus group discussions, reflected further by a reduced consensus regarding *spaciousness*. *Distortion* was an important attribute of audio quality for hearing aid users in this study, and this term was used to capture elements in the sound that a listener feels should not be there. In audio quality and signal processing research, distortion can be an objective characteristic of the signal, and this may hold true in hearing-aid processing of an audio signal and be perceived by listeners; however, given the perceptual focus, participants agreed on this subjective characteristic of *distortion*, which may not necessarily be analogous to objective distortions in a signal. In addition, *harshness* was an attribute linked to sounds (mostly higher in frequency) that are overwhelming or uncomfortable. This attribute may reflect attributes from audio quality research, such as ‘sharpness’, but importantly such experiences might be more prominent and important for listeners with hearing loss. With similarities and potential differences between this work and existing research in mind, it is worth re-iterating that this sensory evaluation process resulted in important perceptual attributes developed by hearing aid users themselves; this was essential in avoiding bias due to the use of existing perceptual attributes or lexicons that may have been derived from research involving people who do not have a hearing loss, and thus may not have been applicable to the lived experiences of listeners with hearing loss.

The second implication for future research is that this work has developed a series of perceptual attributes related to music audio quality, accompanied by working definitions and measurements for empirical use and investigation. It is hoped that these attribute rating scales will be applicable in further research on the music listening experiences of those with hearing loss, and those who use hearing aids or other assistive technologies. There are several key avenues for future work in relation to these attributes. Firstly, it will be imperative to perform further quantitative testing with different listeners to explore the relationships between attributes and overall music audio quality, and potentially in relation to liking or preference, given the known modulation of preference on ratings of technical audio quality (Wilson and Fazenda, 2016). Secondly, the current attribute definitions appeared to reflect the meanings agreed by participants in the focus groups; however, it will be crucial to understand how music listeners naïve to this sensory evaluation process make sense of these details. Finally, these perceptual attributes, although relevant for hearing loss, consider only one example of numerous music listening engagements; as such, future iterations of this sensory evaluation process should be performed to investigate how generalisable the attributes are to other challenging scenarios, such as indoor or outdoor live concert attendance, or during a person’s performing of music.

### 8.3 Limitations

This study has several limitations. To begin, the study involved a sensory panel of 12 participants, with this smaller sample size limiting approaches to quantitative data, for example. However, larger participant samples can introduce complications to sensory evaluation and focus group approaches, and 12 participants was considered appropriate to ensure a focused discussion on audio quality perception, and that different perspectives and voices were contributing equally. There was also a lack of empirical control in terms of online listening tasks, and hearing aid devices used by participants. Sensory panel participants were asked to listen to music samples in two online tasks (individual elicitation and follow-up) over loudspeakers. However, there was certainly variability across participants in terms of playback volume, playback technology, and listening environment. Similarly, participants each used their own hearing aids when listening to the music online and in the focus group sessions. A consequence of this, in relation to audio quality perception, is that experiences may differ for numerous reasons (e.g., different devices, couplings, prescription formulae, processing strategies). However, in the context of this descriptive analysis approach, this was deemed an acceptable compromise for three key reasons: (1) allowing for variability across these levels maximised the opportunity to capture a diverse and extensive perceptual space, ensuring that perceptual attributes were not derived from a narrow set of experiences that failed to capture important aspects; (2) to balance this variability (and what it affords), the descriptive analysis and consensus vocabulary approach adopted in this work allowed participants to navigate variability and diverse perceptual experiences together; (3) ultimately, the variability present across online listening and hearing aid devices reflects an ecologically valid representation of participants’ everyday music listening experiences, meaning that the perceptual attributes are not derived from highly specified experimental conditions or settings. Of course, future iterations of this work may benefit from extended levels of control, where possible and appropriate to the design and participants involved.

Next, it is important to consider how the data are derived from the music samples and processing strategies utilised. A total of 10 music excerpts were taken from the MedleyDB 2.0 database (Bittner et al., 2016), representing various styles and a balance between instrumental music and music with lyrics. Furthermore, much of the sample processing was derived from previous work on hearing aids and music quality (Arehart et al., 2011). However, more work is needed to understand how the perceptual attributes developed by sensory panel participants generalise not only to other listeners and situations, but to broader representations of music. Additionally, processed music samples, such as those that were compressed, would have been doubly processed before being heard by listeners with their hearing aids on. This introduces potential problems in terms of the perceptual experience, but there are two considerations to make: (1) double processing might in fact be commonplace and reflect everyday listening circumstances, for example given pervasive and sometimes heavy compression applied to radio; (2) participants provided terms to describe music in the individual elicitation task twice, once aided and once unaided, with results often being comparable, suggesting that any double processing did not produce anomalous experiences. A further point to note about the sample processing strategies is that these, whilst extreme in some cases, may not have been as detrimental as expected to participants’ listening experiences. For instance, ‘clear’ was the most used term in the individual elicitation task and was also commonly used to describe compressed samples; it is worth highlighting however that this was not the case for bandpass and car noise samples, and that no expectations were established regarding how object-based audio changes would affect experiences of music audio quality. As a closing point regarding the music samples and processing approaches taken, given that these were determined by the researchers, it is possible that any perceptual attribute space is inadvertently influenced by the materials and conditions. Thus, it will be important to consider future study iterations that diversify the music involved, and potentially include participant-selected examples that demonstrate their widest variation in perceptual experience.

A final acknowledgment concerns the diversity of participants’ hearing loss profiles. For sensory evaluation research it has been suggested that, as much as is possible, participants have approximately comparable levels of hearing loss (Zacharov, 2019). This was largely achieved in the current study, with most participants having mild to moderate levels of hearing loss, although there was some variation beyond this range. However, hearing and the perceptual experience of sound is unique to each individual, even within the same ‘level’ or categorization of hearing loss severity. Further to this and beyond hearing loss, each participant has a unique background and set of experiences, evident in the current study at the level of music performance experience for instance. This aural diversity and variability of experience might be sufficiently encapsulated in these processes, and be advantageous for producing impactful insights, especially for the purposes of developing an understanding of the perceptual experiences of music listeners with a hearing loss. Furthermore, understanding key perceptual attributes of audio quality in a heterogenous participant sample is essential for informing future developments and machine learning approaches in signal processing, such that strategies are adaptive and generalizable across many listeners. However, it is essential to be cognizant of the possible limitations involved when working with participants who have diverse hearing loss characteristics. For instance, it is possible that perceptual experiences differ between listeners with mild hearing loss compared to those with more severe loss. Relatedly, producing a consensus from a diverse participant panel may impact which perceptual attributes are central, and which are less important; as a relevant example, it could be that ‘loudness’ is especially pertinent in a panel of participants were severe hearing loss (e.g., in relation to loudness recruitment), but that these group-associated experiences are filtered through the present consensus approach. These considerations are vital motivators for continued sensory evaluation work in this area, in which comparisons across hearing loss severity can be investigated.

## 9 Conclusion

This sensory panel study aimed to develop a foundational understanding of the perceptual experiences of music audio quality for hearing aid users. Through a descriptive analysis approach, twelve participants proposed seven key perceptual attributes of music audio quality, namely *clarity, harshness, distortion, spaciousness, treble strength, middle strength,* and *bass strength* (with an alternative *frequency balance* attribute explored). For each of these attributes, participants also worked with the research team to develop working definitions for each, and create rating scales for future studies. Some of these attributes share similarities with those in audio quality research across different contexts and with listeners who have no hearing impairment; however, some differences may also be apparent, corresponding with issues and problems that music listeners with hearing loss may experience. Hearing aids can have a positive impact on sound quality, even in musical contexts, but more work will be required to address the limitations of such technology and other consumer devices (e.g., those that may develop with object-based audio remixing capabilities), and to develop, improve and innovate in these areas. It is hoped that the present study serves as a first perceptual grounding for these endeavours, and that the attributes are further tested and developed in future work.

## Data availability statement

The original contributions presented in the study are publicly available. This data can be found here: doi: 10.17866/rd.salford.24243637.

## Ethics statement

The studies involving humans were approved by University of Leeds Faculty of Arts, Humanities and Cultures Research Ethics Committee. The studies were conducted in accordance with the local legislation and institutional requirements. The participants provided their written informed consent to participate in this study.

## Author contributions

SB: Conceptualization, Data curation, Formal analysis, Investigation, Methodology, Project administration, Visualization, Writing – original draft, Writing – review & editing. AG: Conceptualization, Data curation, Formal analysis, Funding acquisition, Investigation, Methodology, Project administration, Supervision, Writing – review & editing. TC: Conceptualization, Funding acquisition, Methodology, Project administration, Supervision, Writing – review & editing. MA: Conceptualization, Funding acquisition, Methodology, Supervision, Writing – review & editing. JB: Conceptualization, Funding acquisition, Methodology, Supervision, Writing – review & editing. BF: Conceptualization, Funding acquisition, Methodology, Supervision, Writing – review & editing. JF: Conceptualization, Data curation, Methodology, Writing – review & editing. SG: Conceptualization, Funding acquisition, Writing – review & editing. GR: Conceptualization, Writing – review & editing. RV: Conceptualization, Writing – review & editing. WW: Conceptualization, Funding acquisition, Methodology, Writing – review & editing, Supervision.

## Footnotes

^1^www.musicandhearingaids.org
