# Supplementary material for: Muddy, muddled, or muffled? Understanding the perception of audio quality in music by hearing aid users
Source: Front Psychol. 2024 Feb 21;15:1310176. doi: 10.3389/fpsyg.2024.1310176 (PMC10916511; doi:10.3389/fpsyg.2024.1310176)

**Supplementary Material**

**S1 – Perceptual attribute definitions, shorter definitions, and scale structures**

**Clarity**

*Clarity refers to how well you can hear the different elements of the music, including being able to distinguish between the different sound sources, instruments, or voices in the music, and being able to hear the qualities that distinguish one sound source from another. Unclear music may sound indistinct, mushy, or muddy; clear music may sound clean, distinct, and transparent.*

Short definition:

*Clarity refers to how well you can hear and distinguish between the different instruments and elements within the music.*

Scale endpoints: *very unclear*, and *very clear*

**Harshness**

*Harshness refers to an emphasis or amplification of certain sound qualities (often in the treble frequencies or higher pitches) that can feel overwhelming, abrasive, painful, or discomforting. Harsh sounds may sound piercing, screechy, shrill or sharp.*

Short definition:

*Harshness refers to an uncomfortable overemphasis of certain parts of the sound. It is most often heard in the treble resulting in a piercing, screechy or sharp sounds.*

Scale endpoints: *not harsh*, and *very* *harsh*

**Distortion**

*Distortion refers to a sense that the audio quality of the music contains elements that should not be there, that do not feel right, or that have appeared between the music’s reproduction and your listening of it. These elements may include artefacts (e.g., noise, hiss, pops, crackles), or distortions to pitch (e.g., the pitches sound wrong compared to what you imagine was performed and recorded). No distortion may reflect a sense that the music is an authentic or accurate representation of what was performed and recorded, with no sense of pollution, interference, or distortion in the audio signal.*

Short definition:

*Distortion can be caused by artefacts that shouldn’t be present e.g., noise, hiss, pops or crackles. It can also be caused by the pitches sounding wrong. Music with No distortion sounds like an authentic version of what was performed.*

Scale endpoints: *not distorted*, and *very distorted*

**Spaciousness**

*Imagine hitting a drum in two spaces: a small living room, and then a large cathedral. Whilst your action is the same, the sound produced can take less time or more time to return to silence, as it reverberates in a space.

Spaciousness refers to the perceived presence of these reverberations created by the space in which the music was performed. This may refer to how much you feel the music is ‘coloured’ by this space. A lack of spaciousness may mean that reverberations or a sense of space is not heard, with the opposite true for very spacious sound.*

Short definition:

*Spaciousness refers to how much you feel the music is ‘coloured’ by the performance space, and how much you can hear the reverberations and sense of space.*

Scale endpoints: *Not spacious*, and *very spacious*

**Treble strength**

*Treble strength refers to the perceived strength or prominence of sound qualities that are characterised by higher frequencies in the treble range, or similarly, sounds, instruments or voices with higher pitches.*
Scale endpoints: *Not trebley*, and *very trebley*

**Middle strength**

*Middle strength refers to the perceived strength or prominence of sound qualities that are characterised by middle frequencies found between bass and treble ranges, or similarly, sounds, instruments or voices that pitches perceived as being between lower and higher pitches.*
Scale endpoints: *Not middley*, and *very middley*

**Bass Strength**

*Bass strength refers to the perceived strength or prominence of sound qualities that are characterised by lower frequencies in the bass range, or similarly, sounds, instruments or voices with lower pitches.*
Scale endpoints: *Not bassy*, and *very bassy*

**Frequency balance**

*Frequency balance refers to the perceived, relative balance between treble (or higher pitches of sound) and bass (or lower pitches of sound) in the audio. Audio described as more bassy would be characterised as having stronger or more prominent bass frequencies and pitches in comparison to treble frequencies and pitches, with the opposite true for audio described as more trebley. The middle point of this scale indicates a perceived balance between bass and treble.*

Short definition:

*Frequency balance refers to the perceived balance between treble (or higher pitch) and bass (or lower pitch) sounds.*

Scale endpoints: *very* *bassy,*and *very* *trebley*
Scale midpoint: *balanced*

**Music audio quality**

*Perceived audio quality results from judgments of the sound of the music, in relation to a person’s expectations of how the music should ideally sound to them.*

*Imagine listening to a piece of music in two different ways: listening through a cheap mobile phone, and then listening through high quality loudspeakers. The music is fundamentally same in both cases, but the audio quality is very different.*

Scale endpoints: *very poor,*and *very good*

**S2 – Individual elicitation analysis output (processing conditions, individual participants)**

Most used terms for different sample processing conditions

| **Condition** (*n* samples) | **Most Frequent Terms** (*n* total use) |
| --- | --- |
| Original (10) | *Clear* (61), *Balanced* (18), *Loud* (14) |
| Compression (3) | *Clear* (14), *Balanced* (5), *Blurred** (4) |
| Bandpass Filter (3) | *Loud* (9), *Distorted* (8), *Tinny* (8) |
| Additive Car Noise (3) | *Unclear* (12), *Distorted* (9), *Muffled* (9) |
| Vocals +6dB (1) | *Balanced* (4), *Clear* (4), *Unclear* (4), *Warm* (4) |
| Vocals –6dB (1) | *Clear* (3), *Sharp* (3), *Twangy* (3) |
| Drums +6dB (1) | *Clear* (9), *Thin* (3), *Tinny* (3) |
| Drums –6dB (1) | *Clear* (3), *Spacious* (3), *Echoey** (2) |
| Bass +6dB (1) | *Bassy* (3), *Bright** (2), *Clear** (2) |
| Bass –6dB (1) | *Clear* (3), *Loud* (3), *Balanced** (2) |
| Other +6dB (1) | *Loud* (3), *Brash** (2), *Coherent** (2) |
| Other –6dB (1) | *Clear* (6), *Balanced* (5), *Gentle** (2) |

The five most used terms used by each participant, organized in relation to their hearing loss category, following the WHO 4-frequency average recommendations and categorizations.

| **Hearing Loss Category** | **Participant Number** | **Most Used Terms** | **Total Unique Terms** |
| --- | --- | --- | --- |
| No Impairment | 9 | *Full* (5), *Harsh* (4), *Pale* (4), *Spiky* (4), *Airy* (3) | 114 |
| Mild | 2 | *Balanced* (17), *Clear* (10), *Bassless* (8), *Trebley* (7), *Hard* (6) | 47 |
|  | 8 | *Flat* (4), *Noisy* (4), *Thin* (4), *Blurred* (3), *Bright* (3) | 61 |
| Moderate | 4 | *Narrow* (16), *Muddy* (9), *Unclear* (7), *Compressed* (6), *Bassy* (5) | 58 |
|  | 5 | *Balanced* (8), *Coherent* (8), *Indistinct* (8), *Bassy* (7), *Smooth* (7) | 29 |
|  | 7 | *Clear* (23), *Distant* (15), *Recognisable* (8), *Messy* (7), *Accessible* (2) | 29 |
|  | 11 | *Clear* (5), *Echoey* (5), *Full* (5), *Loud* (5), *Balanced* (4) | 79 |
|  | 12 | *Clear* (25), *Okay* (11), *Good* (10), *Interferency* (7), *Muffled* (7) | 39 |
| Moderately Severe | 1 | *Loud* (26), *Noisy* (15), *Bright* (14), *Clear* (10), *Distorted* (8) | 37 |
|  | 6 | *Clear* (12), *Rhythmic* (10), *Sharp* (10), *Blurred* (8), *Flat* (7) | 47 |
|  | 10 | *Distorted* (19), *Okay* (9), *Unclear* (9), *Clear* (8), *Loud* (7) | 27 |
| Severe | 3 | *Clear* (8), *Defined* (5), *Indistinct* (5), *Mellow* (5), *Clarity* (4) | 25 |

**S3 – Distance matrices for selecting samples in Focus Group 1**
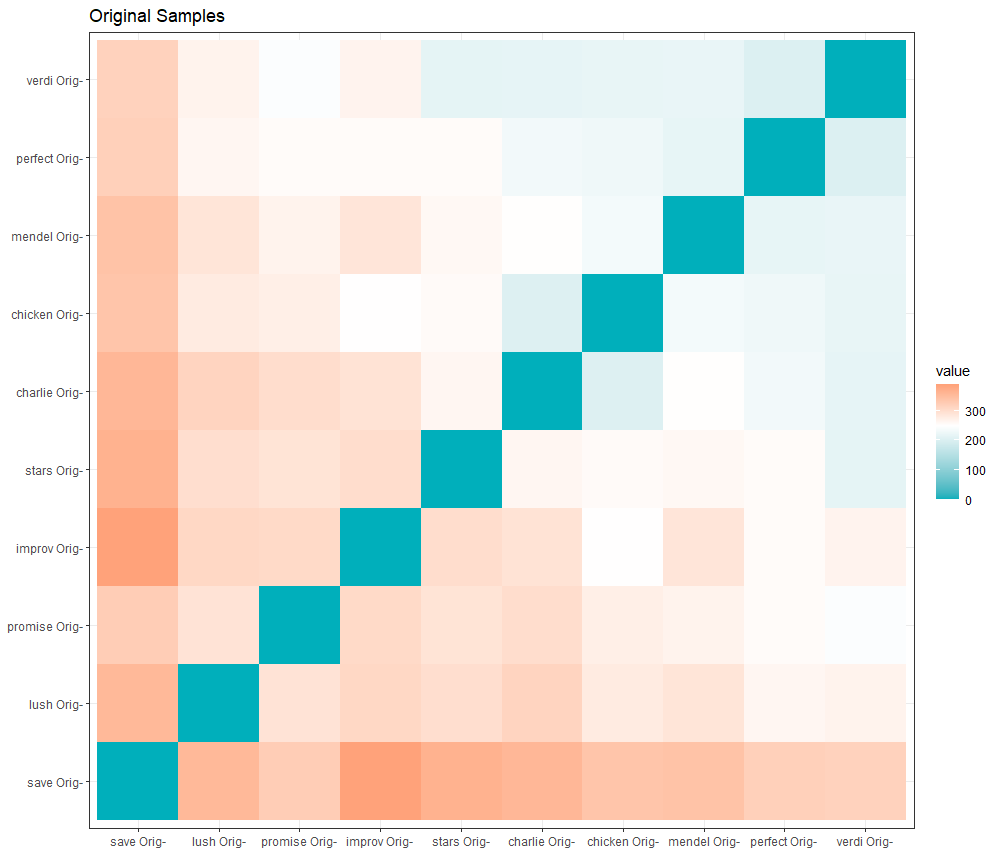


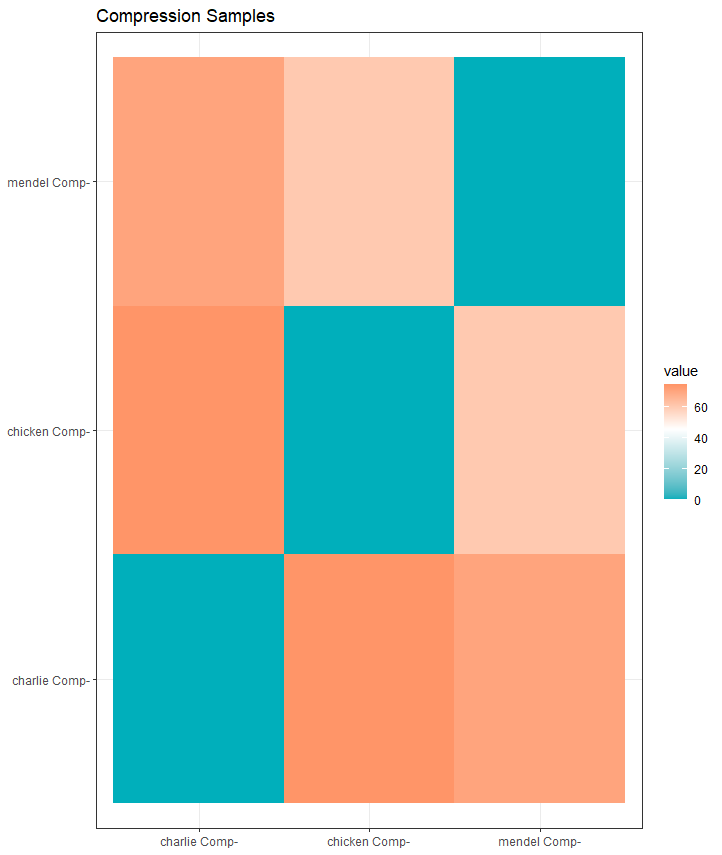


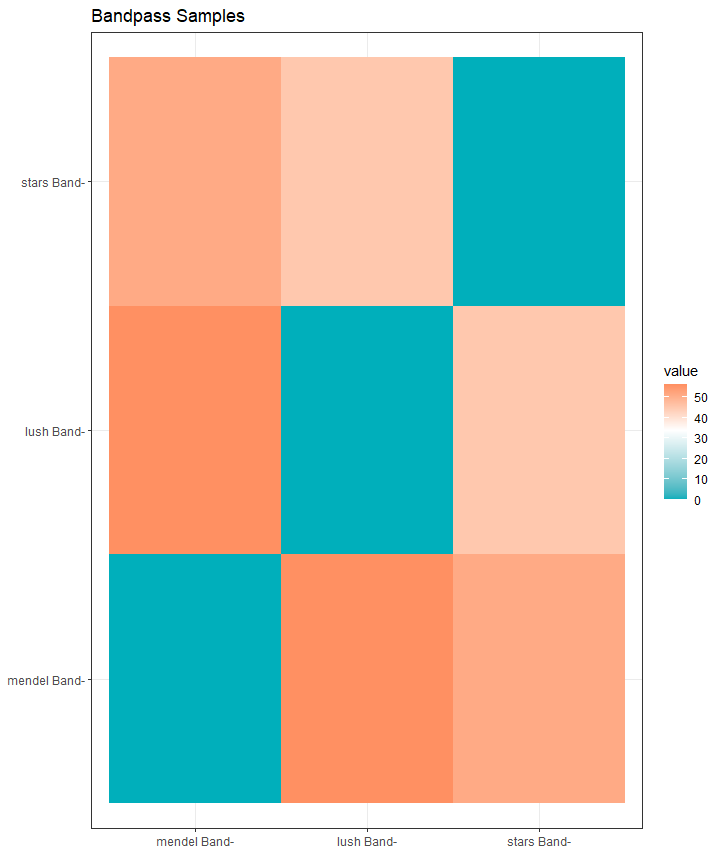


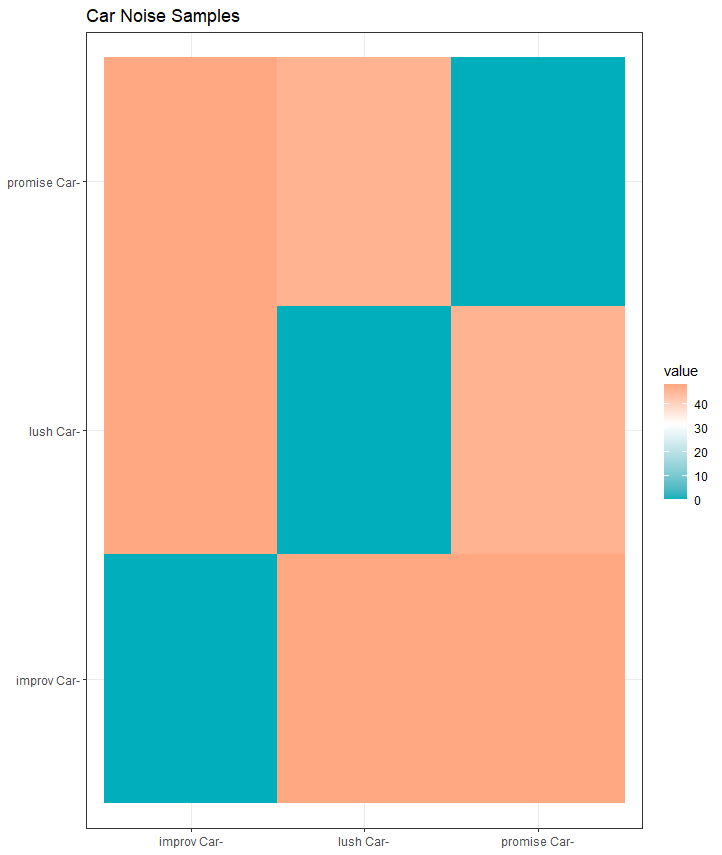


**S4 – Perceptual attribute ratings across 5 music samples in follow-up task**


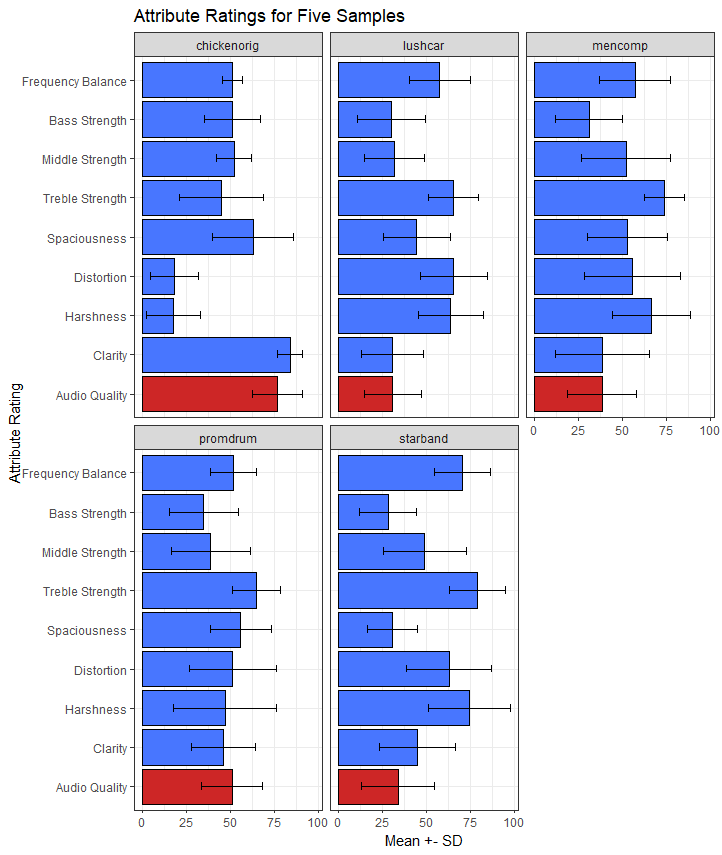

Supplement: Supplementary file 1 [file Data_Sheet_1.docx]
