# Supplementary material for: Muddy, muddled, or muffled? Understanding the perception of audio quality in music by hearing aid users
Source: Front Psychol. 2024 Feb 21;15:1310176. doi: 10.3389/fpsyg.2024.1310176 (PMC10916511; doi:10.3389/fpsyg.2024.1310176)
Supplement: Supplementary file 2 [file Data_Sheet_2.pdf]

Introduction

Sensory Panel Study

Background Questionnaire

In order to understand more about your perception of music with hearing loss, it would be helpful for us to know some detail about participant characteristics. This includes demographics, your history of hearing loss and use of hearing aids, and your musical background and preferences.

We will use this information mainly to describe the characteristics of the sensory panel when we write up the results, but may also use this to explore patterns in the online listening task data. You will not be identified individually on the basis of your responses. All data will be kept confidential as outlined in the Participant Information Sheet for this study.

If you have any questions at all, please do get in touch with us

This survey should take around 10-15 mins to complete.

Please click the arrow to continue.

Please provide your **participant identification number.**

Note - This is the same number you would have used to complete the online listening task previously (you would have originally received this via email for the first online listening task). But any issues with this, please do send us an email.

Demographics

Please provide your age.

What gender do you identify as?

- ○ Male
- ○ Female
- ○ Non-binary / transgender
- ○ Prefer not to say

Please indicate the highest academic qualification you are currently studying or have achieved:

- ○ No qualification
- ○ GCSEs / O-Levels
- ○ A-Levels / Diploma / Baccalaureate / any other final school exam
- ○ Undergraduate degree (e.g., BA, BMus, BSc)
- ○ Postgraduate degree (e.g., MA, MPhil, MMus)
- ○ PhD / DPhil / any other doctorate
- ○ Other

If you have selected 'other' academic qualification, please state it here:

Hearing Loss

Is your hearing loss in:

- ○ One ear
- ○ Both ears

Do you feel that your hearing is noticeably worse in one ear, compared with the other?

- ○ Yes
- ○ No
- ○ Unsure

How would you rate your hearing difficulty <u>without</u> your hearing aids?

Note - If your hearing is noticeably worse in one ear compared to the other, please respond for your <u>best</u> ear.

- ○ No difficulty
- ○ Rarely have difficulty in following / taking part in a conversation
- ○ May have real difficulty following / taking part in a conversation
- ○ Have difficulty hearing and taking part in conversation
- ○ Have great difficulty hearing and taking part in conversation
- ○ Have very great difficulty hearing and taking part in conversation
- ○ Cannot hear any speech

How long have you had hearing loss?

- ○ Less than one year
- ○ 1-5 years
- ○ 5-10 years
- ○ 10-20 years
- ○ More than 20 years
- ○ I do not remember / unsure

When did your hearing loss start?

- ○ Since birth
- ○ When I was a child or teenager
- ○ In my twenties
- ○ In my thirties
- ○ In my forties
- ○ In my fifties
- ○ In my sixties or older
- ○ I do not remember / unsure

How quickly did your hearing loss progress?

- ○ It was a sudden change
- ○ It happened gradually over many months

- It happened gradually over many years
- I do not remember / unsure

Please describe the patterns of your hearing loss as well as you can

- Greater at high frequencies than low frequencies
- Greater at low frequencies than at high frequencies
- Greater at medium than at low or high frequencies
- Almost the same at all frequencies
- Not sure

Tinnitus refers to the perception of noise in your head or ears (such as ringing or buzzing) in the absence of any corresponding source of sound external to your head.

Over the past year, have you had tinnitus in your head or in one or both ears that lasts for more than five minutes at a time?

- Yes, most of the time
- Yes, a lot of the time
- Yes, some of the time
- No, not in the past year
- No, never
- I do not know

Hearing Aids

How long have you worn hearing aids?

- Less than 3 months
- 3 to 6 months
- 6 to 12 months
- 1 to 2 years
- 3 to 4 years
- 5 years or more

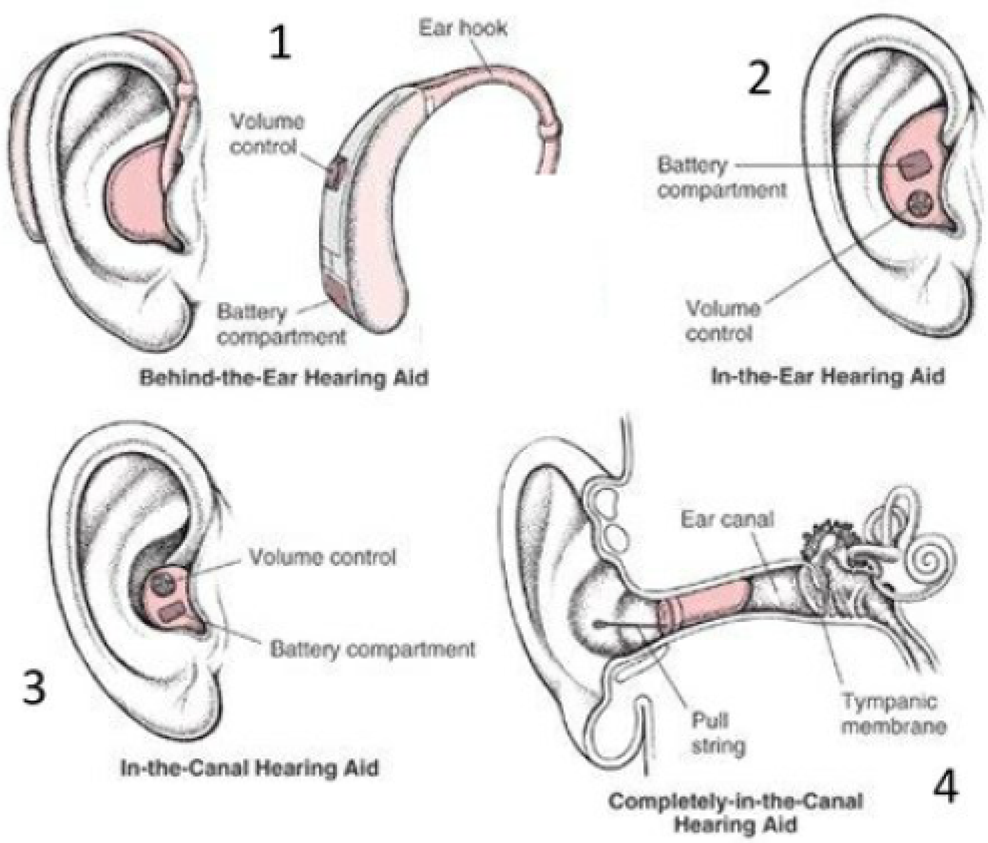

Using the picture above, what type of hearing aids do you use?

- ◯ Behind the ear (BTE) (e. g. with earmould in the ear or with thin tubing and a soft dome)
- ◯ Receiver in the ear (RITE) (sits in the ear canal and the shell of the ear)
- ◯ In the canal (ITC) (the whole hearing aid fits inside the ear canal, but it can be seen)
- ◯ Completely in the canal (CIC) (this fits further into your ear canal than an ITC aid, and is almost invisible)
- ◯ Bone Conducted Hearing Instrument (BCHI) or Bone Anchored Hearing Aid (BAHA)
- ◯ Not sure / Do not know

Open fitting dome

1

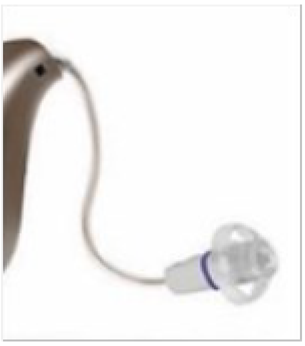

Closed fitting dome

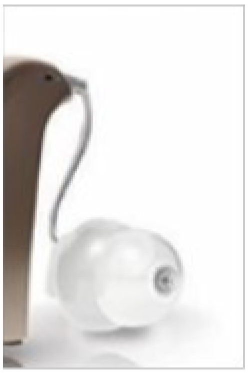

3

Earmould with vent (hole)

2

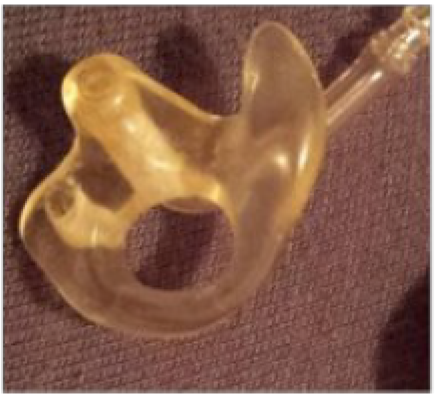

Earmould without vent

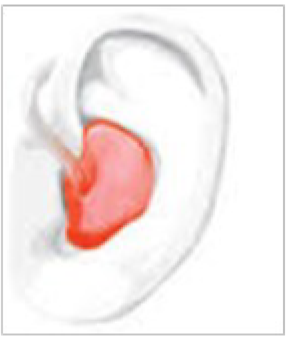

4

Using the pictures above, what dome or mould does your hearing aid have?

- ○ Open: soft dome with openings
- ○ Open: earmould with vent
- ○ Closed: sealed dome
- ○ Closed: earmould with no vent
- ○ Not applicable (e.g., in the canal hearing aid)
- ○ I do not know

Do you have a special program in your hearing aids for music?

- ○ No
- ○ Unsure
- ○ Yes

How frequently do you use your music program?

- ○ Never
- ○ Occasionally

- Sometimes
- Often
- All the time

Using the space below, in a few sentences please feel free to summarise your current experiences of listening to music with hearing aids:

GOLD-MSI

The following questions explore your engagements with music, any musical training your might have received, and your experiences of music.

Importantly, it may be that some of these questions feel difficult or challenging to answer, as some may reflect issues you experience with music listening or performance.

Although it is perhaps unlikely, if at any point you do not feel comfortable or happy with completing these questions, you are of course able to stop at any time.

For each of the following statements, please select the most appropriate category:

	Completely disagree	Strongly disagree	Disagree	Neither agree nor disagree	Agree	Strongly agree	Completely agree
I spend a lot of my free time doing music-related activities.	○	○	○	○	○	○	○
I enjoy writing about music, for example on blogs and forums.	○	○	○	○	○	○	○

	Completely disagree	Strongly disagree	Disagree	Neither agree nor disagree	Agree	Strongly agree	Completely agree
If somebody starts singing a song I don't know, I can usually join in.	○	○	○	○	○	○	○
I can sing or play music from memory.	○	○	○	○	○	○	○
I am able to hit the right notes when I sing along with a recording.	○	○	○	○	○	○	○

	Completely disagree	Strongly disagree	Disagree	Neither agree nor disagree	Agree	Strongly agree	Completely agree
I can compare and discuss differences between two performances or versions of the same piece of music.	○	○	○	○	○	○	○
I have never been complimented for my talents as a musical performer.	○	○	○	○	○	○	○
I often read or search the internet for things related to music.	○	○	○	○	○	○	○
I am not able to sing in harmony when somebody is singing a familiar tune.	○	○	○	○	○	○	○
I am able to identify what is special about a given musical piece.	○	○	○	○	○	○	○

	Completely disagree	Strongly disagree	Disagree	Neither agree nor disagree	Agree	Strongly agree	Completely agree
When I sing, I have no idea whether I'm in tune or not.	○	○	○	○	○	○	○

	Completely disagree	Strongly disagree	Disagree	Neither agree nor disagree	Agree	Strongly agree	Completely agree
Music is kind of an addiction for me - I couldn't live without it.	○	○	○	○	○	○	○
I don’t like singing in public because I’m afraid that I would sing wrong notes.	○	○	○	○	○	○	○
I would not consider myself a musician.	○	○	○	○	○	○	○
After hearing a new song two or three times, I can usually sing it by myself.	○	○	○	○	○	○	○

For this question, please fill in the gap with a number, using the space provided:

I engaged in regular, daily practice of a musical instrument (including voice) for ___ years.

For this question, please fill in the gap with a number, using the space provided:

At the peak of my interest, I practiced ___ hours per day on my primary instrument.

For this question, please fill in the gap with a number, using the space provided:

I can play ___ musical instruments

For this question, please specify a musical instrument (including voice) in the space provided:

The instrument I play best (including voice) is ____

STOMP-R

As a final set of questions, please indicate your basic preference for each of the following genres:

	Dislike strongly	Dislike moderately	Dislike a little	Neither like nor dislike	Like a little	Like moderately	Like strongly
Alternative	○	○	○	○	○	○	○
Bluegrass	○	○	○	○	○	○	○
Blues	○	○	○	○	○	○	○
Classical	○	○	○	○	○	○	○
Country	○	○	○	○	○	○	○
Dance / Electronica	○	○	○	○	○	○	○

	Dislike strongly	Dislike moderately	Dislike a little	Neither like nor dislike	Like a little	Like moderately	Like strongly
Folk	○	○	○	○	○	○	○
Funk	○	○	○	○	○	○	○
Gospel	○	○	○	○	○	○	○
Heavy metal	○	○	○	○	○	○	○
World	○	○	○	○	○	○	○
Jazz	○	○	○	○	○	○	○

	Dislike strongly	Dislike moderately	Dislike a little	Neither like nor dislike	Like a little	Like moderately	Like strongly
New age	○	○	○	○	○	○	○
Opera	○	○	○	○	○	○	○
Pop	○	○	○	○	○	○	○
Punk	○	○	○	○	○	○	○
Rap / hip-hop	○	○	○	○	○	○	○
Reggae	○	○	○	○	○	○	○

	Dislike strongly	Dislike moderately	Dislike a little	Neither like nor dislike	Like a little	Like moderately	Like strongly
Religious	○	○	○	○	○	○	○
Rock	○	○	○	○	○	○	○
Soul / R & B	○	○	○	○	○	○	○

END

Thank you very much for completing this background questionnaire, we really appreciate the time you have given for this!

This now concludes the questionnaire.

As always, if there is any feedback that you would want to give in terms of the questionnaire or the sensory panel study, please do feel free to type your comments in the space provided below.

Powered by Qualtrics
