# Supplementary material for: Muddy, muddled, or muffled? Understanding the perception of audio quality in music by hearing aid users
Source: Front Psychol. 2024 Feb 21;15:1310176. doi: 10.3389/fpsyg.2024.1310176 (PMC10916511; doi:10.3389/fpsyg.2024.1310176)
Supplement: Supplementary file 3 [file Data_Sheet_3.pdf]

Introduction

Sensory Panel - Individual Elicitation

Thank you very much for agreeing to complete this online questionnaire for our sensory panel study. This study aims to identify and understand the perceptual experiences of audio quality during music listening, from the perspective of hearing aid users.

Audio quality may mean different things to different listeners, and we are especially interested in these differences. However for this current questionnaire, you may find it useful to consider a few aspects of audio quality:

1) Imagine listening to the same piece of music across different speakers or headphones, in different locations, or with and without hearing aids - the music itself remains the same, but the sound of the music would be different in your experience. Audio quality refers more to these kinds of variations.

2) Audio quality **does not refer to liking, preference, or enjoyment**

3) Audio quality **goes beyond broad, simple descriptions of quality** (e.g., good or bad); instead, it relates to *attributes* of audio that may be linked to overall descriptions of quality

4) Try to describe audio quality in a way that **feels natural to you**

This **questionnaire should take approximately 1 hour to complete**, and is separated into two parts:

Part 1 - You will be presented with 27 short music samples, and be asked to provide up to three single-word terms that best describe your perceived audio quality of the sample. For this first part, you will be asked to listen to the music with both your hearing aids fitted and turned on, with settings you would normally use for music listening.

Part 2 - Almost identical to the first part, with one main difference - for this second part, you will be asked to listen to the music with your hearing aids removed. Please note that for both parts of the questionnaire, there will be an opportunity to set the volume of the music samples, to something audible and comfortable.

This questionnaire will present one music sample at a time, alongside a space to provide your terms. You can listen to the excerpt as many times as you'd like to help with the task. **However, once you have proceeded to the next music sample, you will not be able to return to previous samples.**

We would encourage you to describe the audio quality of samples not by comparison with previous samples, but in terms of the sample itself (try to avoid using phrases such as '*less* something', or '*more* something').

For this questionnaire, **there are no right or wrong answers in response to any of the music samples**; we are most interested in how you describe what it is you hear!

Please listen to the music samples through a set of loudspeakers, rather than built-in laptop speakers, headphones or earphones.

If you are happy to continue and complete the questionnaire, please click the arrow button below to proceed. If you have any questions or technical difficulties with the questionnaire, do get in touch

Participant Identification

Before we begin, please enter the **participant identification number** that you have been provided over email:

[ ]

Part 1 - Aided Listening

Part One

In this first part of the questionnaire, you will be presented with **27 short music samples**.

Your task will be to listen to each sample, and provide terms that best describe your experience of the sample's **audio quality.**

For these samples, **please listen with both your hearing aids fitted and turned on, with settings you would normally use for music listening**. Unfortunately, if you

would normally stream music directly to your hearing aids, we would ask that this is not used for the current questionnaire.

Please note - if you have any issues with playing back the audio, refreshing the webpage should help to fix this!

When you are ready, please click the arrow button to proceed.

Part 1 - Volume Setting

Before we begin with the first part, please listen to the following piece of music, and use this to **adjust your volume/playback to a level that you can hear, and that is comfortable.**

Please click the play button below to hear the music.

0:00 / 0:20

Once you are happy with the volume levels, please proceed to the first music sample by clicking the arrow button below to proceed.

aid_charlieOrig

Please listen to the following music sample, focussing on its **audio quality**.

Feel free to listen as many times as needed. To listen, please click the play button below.

0:00 / 0:15

Please provide up to three **single-word** terms that best describe the **audio quality** of the music just heard:

Term 1

Term 2

Term 3

aid_chickenOrig

Please listen to the following music sample, focussing on its **audio quality**.

Feel free to listen as many times as needed. To listen, please click the play button below.

0:00 / 0:14

Please provide up to three **single-word** terms that best describe the **audio quality** of the music just heard:

Term 1	
Term 2	
Term 3	

aid_improvOrig

Please listen to the following music sample, focussing on its **audio quality**.

Feel free to listen as many times as needed. To listen, please click the play button below.

0:00 / 0:15

Please provide up to three **single-word** terms that best describe the **audio quality** of the music just heard:

Term 1	
Term 2	
Term 3	

aid_lushOrig

Please listen to the following music sample, focussing on its **audio quality**.

Feel free to listen as many times as needed. To listen, please click the play button below.

0:00 / 0:16

Please provide up to three **single-word** terms that best describe the **audio quality** of the music just heard:

Term 1	
Term 2	
Term 3	

aid_mendelOrig

Please listen to the following music sample, focussing on its **audio quality**.

Feel free to listen as many times as needed. To listen, please click the play button below.

0:00 / 0:17

Please provide up to three **single-word** terms that best describe the **audio quality** of the music just heard:

Term 1	
Term 2	
Term 3	

aid_perfectOrig

Please listen to the following music sample, focussing on its **audio quality**.

Feel free to listen as many times as needed. To listen, please click the play button below.

0:00 / 0:16

Please provide up to three **single-word** terms that best describe the **audio quality** of the music just heard:

Term 1

Term 2

Term 3

aid_promiseOrig

Please listen to the following music sample, focussing on its **audio quality**.

Feel free to listen as many times as needed. To listen, please click the play button below.

0:00 / 0:12

Please provide up to three **single-word** terms that best describe the **audio quality** of the music just heard:

Term 1

Term 2

Term 3

aid_saveOrig

Please listen to the following music sample, focussing on its **audio quality**.

Feel free to listen as many times as needed. To listen, please click the play button below.

0:00 / 0:12

Please provide up to three **single-word** terms that best describe the **audio quality** of the music just heard:

Term 1

Term 2

Term 3

aid_starsOrig

Please listen to the following music sample, focussing on its **audio quality**.

Feel free to listen as many times as needed. To listen, please click the play button below.

0:00 / 0:15

Please provide up to three **single-word** terms that best describe the **audio quality** of the music just heard:

Term 1

Term 2

Term 3

aid_verdiOrig

Please listen to the following music sample, focussing on its **audio quality**.

Feel free to listen as many times as needed. To listen, please click the play button below.

0:00 / 0:13

Please provide up to three **single-word** terms that best describe the **audio quality** of the music just heard:

Term 1

Term 2

Term 3

aid_improvCar

Please listen to the following music sample, focussing on its **audio quality**.

Feel free to listen as many times as needed. To listen, please click the play button below.

0:00 / 0:15

Please provide up to three **single-word** terms that best describe the **audio quality** of the music just heard:

Term 1

Term 2

Term 3

aid_lushCar

Please listen to the following music sample, focussing on its **audio quality**.

Feel free to listen as many times as needed. To listen, please click the play button below.

0:00 / 0:00

Please provide up to three **single-word** terms that best describe the **audio quality** of the music just heard:

Term 1

Term 2

Term 3

aid_promiseCar

Please listen to the following music sample, focussing on its **audio quality**.

Feel free to listen as many times as needed. To listen, please click the play button below.

0:00 / 0:00

Please provide up to three **single-word** terms that best describe the **audio quality** of the music just heard:

Term 1
Term 2
Term 3

aid_charlieComp

Please listen to the following music sample, focussing on its **audio quality**.

Feel free to listen as many times as needed. To listen, please click the play button below.

0:00 / 0:00

Please provide up to three **single-word** terms that best describe the **audio quality** of the music just heard:

Term 1
Term 2
Term 3

aid_chickenComp

Please listen to the following music sample, focussing on its **audio quality**.

Feel free to listen as many times as needed. To listen, please click the play button below.

0:00 / 0:00

Please provide up to three **single-word** terms that best describe the **audio quality** of the music just heard:

Term 1
Term 2
Term 3

aid_mendelComp

Please listen to the following music sample, focussing on its **audio quality**.

Feel free to listen as many times as needed. To listen, please click the play button below.

0:00 / 0:00

Please provide up to three **single-word** terms that best describe the **audio quality** of the music just heard:

Term 1

Term 2

Term 3

aid_lushBand

Please listen to the following music sample, focussing on its **audio quality**.

Feel free to listen as many times as needed. To listen, please click the play button below.

0:00 / 0:00

Please provide up to three **single-word** terms that best describe the **audio quality** of the music just heard:

Term 1

Term 2

Term 3

aid_mendelBand

Please listen to the following music sample, focussing on its **audio quality**.

Feel free to listen as many times as needed. To listen, please click the play button below.

0:00 / 0:00

Please provide up to three **single-word** terms that best describe the **audio quality** of the music just heard:

Term 1	
Term 2	
Term 3	

aid_starsBand

Please listen to the following music sample, focussing on its **audio quality**.

Feel free to listen as many times as needed. To listen, please click the play button below.

0:00 / 0:00

Please provide up to three **single-word** terms that best describe the **audio quality** of the music just heard:

Term 1	
Term 2	
Term 3	

aid_lushVox-

Please listen to the following music sample, focussing on its **audio quality**.

Feel free to listen as many times as needed. To listen, please click the play button below.

0:00 / 0:00

Please provide up to three **single-word** terms that best describe the **audio quality** of the music just heard:

Term 1

Term 2

Term 3

aid_lushOther+

Please listen to the following music sample, focussing on its **audio quality**.

Feel free to listen as many times as needed. To listen, please click the play button below.

0:00 / 0:00

Please provide up to three **single-word** terms that best describe the **audio quality** of the music just heard:

Term 1

Term 2

Term 3

aid_perfectOther-

Please listen to the following music sample, focussing on its **audio quality**.

Feel free to listen as many times as needed. To listen, please click the play button below.

0:00 / 0:00

Please provide up to three **single-word** terms that best describe the **audio quality** of the music just heard:

Term 1

Term 2

Term 3

aid_perfectBass+

Please listen to the following music sample, focussing on its **audio quality**.

Feel free to listen as many times as needed. To listen, please click the play button below.

0:00 / 0:00

Please provide up to three **single-word** terms that best describe the **audio quality** of the music just heard:

Term 1

Term 2

Term 3

aid_promiseVox+

Please listen to the following music sample, focussing on its **audio quality**.

Feel free to listen as many times as needed. To listen, please click the play button below.

0:00 / 0:00

Please provide up to three **single-word** terms that best describe the **audio quality** of the music just heard:

Term 1

Term 2

Term 3

aid_promiseDrum-

Please listen to the following music sample, focussing on its **audio quality**.

Feel free to listen as many times as needed. To listen, please click the play button below.

0:00 / 0:00

Please provide up to three **single-word** terms that best describe the **audio quality** of the music just heard:

Term 1	
Term 2	
Term 3	

aid_starsBass-

Please listen to the following music sample, focussing on its **audio quality**.

Feel free to listen as many times as needed. To listen, please click the play button below.

0:00 / 0:00

Please provide up to three **single-word** terms that best describe the **audio quality** of the music just heard:

Term 1	
Term 2	
Term 3	

aid_starsDrum+

Please listen to the following music sample, focussing on its **audio quality**.

Feel free to listen as many times as needed. To listen, please click the play button below.

0:00 / 0:00

Please provide up to three **single-word** terms that best describe the **audio quality** of the music just heard:

Term 1

Term 2

Term 3

Part Two - Unaided Listening

<u>**Part Two**</u>

At this point, please feel free to take a short break or rest!

What follows is the second part of this questionnaire. The task remains almost identical.

You will again be presented with **27 short music samples**, and your task will be to listen to each sample, and provide terms that best describe your experience of the **sample's audio quality.**

There is **one main difference** - **please listen to the following music samples with your hearing aids removed.**

Quick reminder - if you have any issues with playing back the audio, refreshing the webpage should help to fix this!

When you are ready, please click the arrow button to proceed, where you can check your volume/playback levels.

Part 2 - Volume Setting

Before starting the 2nd part of the questionnaire, please listen to the following piece of music **without your hearing aids, and adjust your volume/playback to a level that you can hear, and that is comfortable.**

Please click the play button below to hear the music.

0:00 / 0:00

Once you are happy with the volume levels, please proceed to the next music sample by clicking the arrow button below to proceed.

un_charlieOrig

Please listen to the following music sample, focussing on its **audio quality**.

Feel free to listen as many times as needed. To listen, please click the play button below.

0:00 / 0:00

Please provide up to three **single-word** terms that best describe the **audio quality** of the music just heard:

Term 1

Term 2

Term 3

un_chickenOrig

Please listen to the following music sample, focussing on its **audio quality**.

Feel free to listen as many times as needed. To listen, please click the play button below.

0:00 / 0:00

Please provide up to three **single-word** terms that best describe the **audio quality** of the music just heard:

Term 1

Term 2

Term 3

un_improvOrig

Please listen to the following music sample, focussing on its **audio quality**.

Feel free to listen as many times as needed. To listen, please click the play button

below.

0:00 / 0:00

Please provide up to three **single-word** terms that best describe the **audio quality** of the music just heard:

Term 1

Term 2

Term 3

un_lushOrig

Please listen to the following music sample, focussing on its **audio quality**.

Feel free to listen as many times as needed. To listen, please click the play button below.

0:00 / 0:00

Please provide up to three **single-word** terms that best describe the **audio quality** of the music just heard:

Term 1

Term 2

Term 3

un_mendelOrig

Please listen to the following music sample, focussing on its **audio quality**.

Feel free to listen as many times as needed. To listen, please click the play button below.

0:00 / 0:00

Please provide up to three **single-word** terms that best describe the **audio quality** of

the music just heard:

Term 1

Term 2

Term 3

un_perfectOrig

Please listen to the following music sample, focussing on its **audio quality**.

Feel free to listen as many times as needed. To listen, please click the play button below.

0:00 / 0:00

Please provide up to three **single-word** terms that best describe the **audio quality** of the music just heard:

Term 1

Term 2

Term 3

un_promiseOrig

Please listen to the following music sample, focussing on its **audio quality**.

Feel free to listen as many times as needed. To listen, please click the play button below.

0:00 / 0:00

Please provide up to three **single-word** terms that best describe the **audio quality** of the music just heard:

Term 1

Term 2

Term 3

un_saveOrig

Please listen to the following music sample, focussing on its **audio quality**.

Feel free to listen as many times as needed. To listen, please click the play button below.

0:00 / 0:00

Please provide up to three **single-word** terms that best describe the **audio quality** of the music just heard:

Term 1

Term 2

Term 3

un_starsOrig

Please listen to the following music sample, focussing on its **audio quality**.

Feel free to listen as many times as needed. To listen, please click the play button below.

0:00 / 0:00

Please provide up to three **single-word** terms that best describe the **audio quality** of the music just heard:

Term 1

Term 2

Term 3

un_verdiOrig

Please listen to the following music sample, focussing on its **audio quality**.

Feel free to listen as many times as needed. To listen, please click the play button below.

0:00 / 0:00

Please provide up to three **single-word** terms that best describe the **audio quality** of the music just heard:

Term 1

Term 2

Term 3

un_improvCar

Please listen to the following music sample, focussing on its **audio quality**.

Feel free to listen as many times as needed. To listen, please click the play button below.

0:00 / 0:00

Please provide up to three **single-word** terms that best describe the **audio quality** of the music just heard:

Term 1

Term 2

Term 3

un_lushCar

Please listen to the following music sample, focussing on its **audio quality**.

Feel free to listen as many times as needed. To listen, please click the play button below.

0:00 / 0:00

Please provide up to three **single-word** terms that best describe the **audio quality** of the music just heard:

Term 1

Term 2

Term 3

un_promiseCar

Please listen to the following music sample, focussing on its **audio quality**.

Feel free to listen as many times as needed. To listen, please click the play button below.

0:00 / 0:00

Please provide up to three **single-word** terms that best describe the **audio quality** of the music just heard:

Term 1

Term 2

Term 3

un_charlieComp

Please listen to the following music sample, focussing on its **audio quality**.

Feel free to listen as many times as needed. To listen, please click the play button below.

0:00 / 0:00

Please provide up to three **single-word** terms that best describe the **audio quality** of the music just heard:

Term 1

Term 2

Term 3

un_chickenComp

Please listen to the following music sample, focussing on its **audio quality**.

Feel free to listen as many times as needed. To listen, please click the play button below.

0:00 / 0:00

Please provide up to three **single-word** terms that best describe the **audio quality** of the music just heard:

Term 1

Term 2

Term 3

un_mendelComp

Please listen to the following music sample, focussing on its **audio quality**.

Feel free to listen as many times as needed. To listen, please click the play button below.

0:00 / 0:00

Please provide up to three **single-word** terms that best describe the **audio quality** of the music just heard:

Term 1

Term 2

Term 3

un_lushBand

Please listen to the following music sample, focussing on its **audio quality**.

Feel free to listen as many times as needed. To listen, please click the play button below.

0:00 / 0:00

Please provide up to three **single-word** terms that best describe the **audio quality** of the music just heard:

Term 1

Term 2

Term 3

un_mendelBand

Please listen to the following music sample, focussing on its **audio quality**.

Feel free to listen as many times as needed. To listen, please click the play button below.

0:00 / 0:00

Please provide up to three **single-word** terms that best describe the **audio quality** of the music just heard:

Term 1

Term 2

Term 3

un_starsBand

Please listen to the following music sample, focussing on its **audio quality**.

Feel free to listen as many times as needed. To listen, please click the play button below.

0:00 / 0:00

Please provide up to three **single-word** terms that best describe the **audio quality** of the music just heard:

Term 1

Term 2

Term 3

un_lushVox-

Please listen to the following music sample, focussing on its **audio quality**.

Feel free to listen as many times as needed. To listen, please click the play button below.

0:00 / 0:00

Please provide up to three **single-word** terms that best describe the **audio quality** of the music just heard:

Term 1

Term 2

Term 3

un_lushOther+

Please listen to the following music sample, focussing on its **audio quality**.

Feel free to listen as many times as needed. To listen, please click the play button below.

0:00 / 0:00

Please provide up to three **single-word** terms that best describe the **audio quality** of the music just heard:

Term 1

Term 2

Term 3

un_perfectOther-

Please listen to the following music sample, focussing on its **audio quality**.

Feel free to listen as many times as needed. To listen, please click the play button below.

0:00 / 0:00

Please provide up to three **single-word** terms that best describe the **audio quality** of the music just heard:

Term 1

Term 2

Term 3

un_perfectBass+

Please listen to the following music sample, focussing on its **audio quality**.

Feel free to listen as many times as needed. To listen, please click the play button below.

0:00 / 0:00

Please provide up to three **single-word** terms that best describe the **audio quality** of the music just heard:

Term 1

Term 2

Term 3

un_promiseVox+

Please listen to the following music sample, focussing on its **audio quality**.

Feel free to listen as many times as needed. To listen, please click the play button below.

0:00 / 0:00

Please provide up to three **single-word** terms that best describe the **audio quality** of the music just heard:

Term 1	
Term 2	
Term 3	

un_promiseDrum-

Please listen to the following music sample, focussing on its **audio quality**.

Feel free to listen as many times as needed. To listen, please click the play button below.

0:00 / 0:00

Please provide up to three **single-word** terms that best describe the **audio quality** of the music just heard:

Term 1	
Term 2	
Term 3	

un_starsBass-

Please listen to the following music sample, focussing on its **audio quality**.

Feel free to listen as many times as needed. To listen, please click the play button below.

0:00 / 0:00

Please provide up to three **single-word** terms that best describe the **audio quality** of the music just heard:

Term 1

Term 2

Term 3

un_starsDrum+

Please listen to the following music sample, focussing on its **audio quality**.

Feel free to listen as many times as needed. To listen, please click the play button below.

0:00 / 0:00

Please provide up to three **single-word** terms that best describe the **audio quality** of the music just heard:

Term 1

Term 2

Term 3

Finish and Feedback

Thank you very much for generously giving up your time to complete this online questionnaire.

This now concludes the questionnaire.

We would be very interested in **any comments, feedback or suggestions** that you may have regarding this questionnaire, whether that is in terms of the information provided, the music samples, the task itself, or any other aspects.

Please feel free to leave any comments in the space below.

To end the questionnaire, please click the arrow below. And thank you so much again! We really look forward to discussing the ways to describe audio quality, in our upcoming focus group discussions.

Powered by Qualtrics
