# Supplementary material for: Muddy, muddled, or muffled? Understanding the perception of audio quality in music by hearing aid users
Source: Front Psychol. 2024 Feb 21;15:1310176. doi: 10.3389/fpsyg.2024.1310176 (PMC10916511; doi:10.3389/fpsyg.2024.1310176)
Supplement: Supplementary file 4 [file Data_Sheet_4.pdf]

Introduction to Follow-Up

<u>**Sensory Panel**</u>

Final Follow-Up Task

Hello, and thank you very much for your exceptional work, and incredible commitment during this sensory panel study; once again, we really appreciate this.

After three focus groups, you and other members of the sensory panel developed, through consensus and agreement, perceptual attributes of audio quality that are important in your experiences of listening to music. You also worked together to provide single-word labels for these attributes, to provide rough definitions for these attributes, and to outline the broad structure of rating scales that could be used to capture these attributes.

After our 3rd focus group, we listened through and analysed the discussions of the group, and have attempted to generate some short definitions for each of the attributes you have developed.

With these definitions, we aimed to:

1) Capture the overall meaning of the attribute as discussed and agreed by the sensory panel members

2) Keep language simple and definitions short, to make sure that new music listeners

can understand the attributes

This follow-up task is an opportunity for you to see these definitions we have written, to try out the attribute scales you developed to rate some music samples, and to provide any feedback on how well our definitions captured the meaning agreed by the sensory panel members.

The task is structured as follows:

1) You will be presented with the perceptual attribute definitions we have written

2) You will then use your perceptual attribute scales to rate some music samples

3) You will be presented again with the perceptual attribute definitions, and be asked to provide feedback on their suitability and usability

This follow-up task is optional, but we hope that you would find it interesting and insightful to use the perceptual attribute scales that you have directly developed!

Your responses will be used to refine these perceptual attribute scales; you will not be identified individually on the basis of your responses in subsequent work. All data will be kept confidential as outlined in the Participant Information Sheet for the sensory panel study.

This task is designed to work best on a computer screen, rather than a phone or tablet - if possible, it may be best to complete this on a computer.

If you have any questions at all, please do get in touch with us

This task should take no more than 45 minutes to complete.

To proceed please click the arrow below.

Participant Identification

Please provide your **participant identification number**.

Note - This is the same number you would have used to complete the online listening task previously (you would have originally received this via email for the first online listening task). But any issues with this, please do send us an email.

Definitions Overview

Perceptual Attributes - Definitions

On the following screen, you will be presented with definitions that have been proposed to capture the perceptual attributes you developed, and the meaning of these as discussed by the sensory panel group. After reading these definitions, you will be asked to rate some music samples with the attribute scales you developed.

Please note - as a group, you agreed that 3 scales might best capture the attribute of 'frequency balance' (e.g., treble, middle, and bass). However, this may be too many scales for some future studies to incorporate. As such, we have also proposed an additional definition which hopefully captures an alternative one-scale option for 'frequency balance', that the group appeared to understand intuitively during the focus groups. We would welcome your feedback on the 3 individual scales, and also this one alternative scale. Thank you.

Please proceed by clicking the arrow below.

Please see the following proposed definition for the perceptual attribute that you as a group have labelled **clarity**:

Clarity refers to how well you can hear the different elements of the music, including being able to distinguish between the different sound sources, instruments, or voices in the music, and being able to hear the qualities that distinguish one sound source from another. Unclear music may sound indistinct, mushy, or muddy; clear music may sound clean, distinct, and transparent.

Short definition:

Clarity refers to how well you can hear and distinguish between the different instruments and elements within the music.

Scale endpoints: *very unclear*, and *very clear*

Please see the following proposed definition for the perceptual attribute that you as a group have labelled **harshness**:

Harshness refers to an emphasis or amplification of certain sound qualities (often in the treble frequencies or higher pitches) that can feel overwhelming, abrasive, painful, or discomforting. Harsh sounds may sound piercing, screechy, shrill or sharp.

Short definition:

Harshness refers to an uncomfortable overemphasis of certain parts of the sound. It is most often heard in the treble resulting in a piercing, screechy or sharp sounds.

Scale endpoints: *not harsh*, and *very harsh*

Please see the following proposed definition for the perceptual attribute that you as a group have labelled **distortion**:

Distortion refers to a sense that the audio quality of the music contains elements that

should not be there, that do not feel right, or that have appeared between the music's reproduction and your listening of it. These elements may include artefacts (e.g., noise, hiss, pops, crackles), or distortions to pitch (e.g., the pitches sound wrong compared to what you imagine was performed and recorded). No distortion may reflect a sense that the music is an authentic or accurate representation of what was performed and recorded, with no sense of pollution, interference, or distortion in the audio signal.

Short definition:

Distortion can be caused by artefacts that shouldn't be present e.g., noise, hiss, pops or crackles. It can also be caused by the pitches sounding wrong. Music with No distortion sounds like an authentic version of what was performed.

Scale endpoints: *not distorted*, and *very distorted*

Please see the following proposed definition for the perceptual attribute that you as a group have labelled **spaciousness**:

Imagine hitting a drum in two spaces: a small living room, and then a large cathedral. Whilst your action is the same, the sound produced can take less time or more time to return to silence, as it reverberates in a space.

Spaciousness refers to the perceived presence of these reverberations created by the space in which the music was performed. This may refer to how much you feel the music is 'coloured' by this space. A lack of spaciousness may mean that reverberations or a sense of space is not heard, with the opposite true for very spacious sound.

Short definition:

Spaciousness refers to how much you feel the music is 'coloured' by the performance space, and how much you can hear the reverberations and sense of space.

Scale endpoints: *Not spacious*, and *very spacious*

Please see the following proposed definition for the perceptual attribute that you as a group have labelled **treble strength**:

Treble strength refers to the perceived strength or prominence of sound qualities that are characterised by higher frequencies in the treble range, or similarly, sounds, instruments or voices with higher pitches.

Scale endpoints: *Not trebley*, and *very trebley*

Please see the following proposed definition for the perceptual attribute that you as a group have labelled **middle strength**:

Middle strength refers to the perceived strength or prominence of sound qualities that are characterised by middle frequencies found between bass and treble ranges, or similarly, sounds, instruments or voices that pitches perceived as being between lower and higher pitches.

Scale endpoints: *Not middley*, and *very middley*

Please see the following proposed definition for the perceptual attribute that you as a group have labelled **bass strength**:

Bass strength refers to the perceived strength or prominence of sound qualities that are characterised by lower frequencies in the bass range, or similarly, sounds, instruments or voices with lower pitches.

Scale endpoints: *Not bassy*, and *very bassy*

Please see the following proposed definition for the perceptual attribute of **frequency balance**:

Frequency balance refers to the perceived, relative balance between treble (or higher pitches of sound) and bass (or lower pitches of sound) in the audio. Audio described as more bassy would be characterised as having stronger or more prominent bass frequencies and pitches in comparison to treble frequencies and pitches, with the opposite true for audio described as more trebley. The middle point of this scale indicates a perceived balance between bass and treble.

Short definition:

Frequency balance refers to the perceived balance between treble (or higher pitch) and bass (or lower pitch) sounds.

Scale endpoints: *very bassy*, and *very trebley*
Scale midpoint: *balanced*

Please see the following proposed definition for **overall audio quality**:

Perceived audio quality results from judgments of the sound of the music, in relation to a person's expectations of how the music should ideally sound to them.

Imagine listening to a piece of music in two different ways: listening through a cheap mobile phone, and then listening through high quality loudspeakers. The music is fundamentally same in both cases, but the audio quality is very different.

Scale endpoints: *very poor*, and *very good*

Music Samples Overview

Perceptual Attributes - Rating Music Samples

You will now be asked to use the perceptual attribute scales you have developed to rate 5 short music samples.

You can listen to these samples as many times as needed to do this.

Please note - when using the scales, do try your best to use them consistently in a way that reflects the definitions.

If you ever need a reminder of the current definitions, you can hover your mouse over the perceptual attribute label, which is highlighted in **blue**.

To proceed please click the arrow below.

Mendelssohn Comp

Please listen to the music sample above, and rate the audio quality of this using the attribute scales below.

To make it easier to replay the music, this sample will remain at the top of your screen as you scroll.

For a reminder of attribute definitions, please hover your mouse over the attribute titles coloured in **blue**.

You can listen to the sample as many times as you need to.

Clarity

Very unclear										Very clear
0	10	20	30	40	50	60	70	80	90	100

-

Harshness

Not harsh										Very harsh
0	10	20	30	40	50	60	70	80	90	100

-

Distortion

Not distorted										Very distorted
0	10	20	30	40	50	60	70	80	90	100

-

Spaciousness

Dead, not spacious — Live, very spacious

0 10 20 30 40 50 60 70 80 90 100

-

Treble Strength

Not trebley — Very trebley

0 10 20 30 40 50 60 70 80 90 100

-

Middle Strength

Not middley — Very middley

0 10 20 30 40 50 60 70 80 90 100

-

Bass Strength

Not bassy — Very bassy

0 10 20 30 40 50 60 70 80 90 100

-

Frequency Balance

Very bassy Balanced Very trebley

0 10 20 30 40 50 60 70 80 90 100

-

Overall Audio Quality

Very poor Very good

0 10 20 30 40 50 60 70 80 90 100

-

Lush Car

Please listen to the music sample above, and rate the audio quality of this using the attribute scales below.

To make it easier to replay the music, this sample will remain at the top of your screen as you scroll.

For a reminder of attribute definitions, please hover your mouse over the attribute titles coloured in **blue**.

You can listen to the sample as many times as you need to.

Clarity

Very unclear | Very clear

0 10 20 30 40 50 60 70 80 90 100

-

Harshness

Not harsh | Very harsh

0 10 20 30 40 50 60 70 80 90 100

-

Distortion

Not distorted | Very distorted

0 10 20 30 40 50 60 70 80 90 100

-

Spaciousness

Dead, not spacious Live, very spacious

0 10 20 30 40 50 60 70 80 90 100

-

Treble Strength

Not trebley Very trebley

0 10 20 30 40 50 60 70 80 90 100

-

Middle Strength

Not middley Very middley

0 10 20 30 40 50 60 70 80 90 100

-

Bass Strength

Not bassy Very bassy

0 10 20 30 40 50 60 70 80 90 100

-

Frequency Balance

Very bassy					Balanced				Very trebley	
0	10	20	30	40	50	60	70	80	90	100

-

Overall Audio Quality

Very poor									Very good	
0	10	20	30	40	50	60	70	80	90	100

-

Promise Drum-

Please listen to the music sample above, and rate the audio quality of this using the attribute scales below.

To make it easier to replay the music, this sample will remain at the top of your screen as you scroll.

For a reminder of attribute definitions, please hover your mouse over the attribute titles coloured in **blue**.

You can listen to the sample as many times as you need to.

Clarity

Very unclear										Very clear
0	10	20	30	40	50	60	70	80	90	100

-

Harshness

Not harsh										Very harsh
0	10	20	30	40	50	60	70	80	90	100

-

Distortion

Not distorted										Very distorted
0	10	20	30	40	50	60	70	80	90	100

-

Spaciousness

Dead, not spacious										Live, very spacious
0	10	20	30	40	50	60	70	80	90	100

-

Treble Strength

Not trebley Very trebley

0 10 20 30 40 50 60 70 80 90 100

-

Middle Strength

Not middley Very middley

0 10 20 30 40 50 60 70 80 90 100

-

Bass Strength

Not bassy Very bassy

0 10 20 30 40 50 60 70 80 90 100

-

Frequency Balance

Very bassy Balanced Very trebley

0 10 20 30 40 50 60 70 80 90 100

-

Overall Audio Quality

Very poor Very good

0 10 20 30 40 50 60 70 80 90 100

-

Stars Band

Please listen to the music sample above, and rate the audio quality of this using the attribute scales below.

To make it easier to replay the music, this sample will remain at the top of your screen as you scroll.

For a reminder of attribute definitions, please hover your mouse over the attribute titles coloured in **blue**.

You can listen to the sample as many times as you need to.

Clarity

Very unclear Very clear

0 10 20 30 40 50 60 70 80 90 100

-

Harshness

Not harsh Very harsh

0 10 20 30 40 50 60 70 80 90 100

-

Distortion

Not distorted Very distorted

0 10 20 30 40 50 60 70 80 90 100

-

Spaciousness

Dead, not spacious Live, very spacious

0 10 20 30 40 50 60 70 80 90 100

-

Treble Strength

Not trebley Very trebley

0 10 20 30 40 50 60 70 80 90 100

-

Middle Strength

Not middley Very middley

0 10 20 30 40 50 60 70 80 90 100

-

Bass Strength

Not bassy Very bassy

0 10 20 30 40 50 60 70 80 90 100

-

Frequency Balance

Very bassy Balanced Very trebley

0 10 20 30 40 50 60 70 80 90 100

-

Overall Audio Quality

Very poor Very good

0 10 20 30 40 50 60 70 80 90 100

-

Chicken Original

Please listen to the music sample above, and rate the audio quality of this using the attribute scales below.

To make it easier to replay the music, this sample will remain at the top of your screen as you scroll.

For a reminder of attribute definitions, please hover your mouse over the attribute titles coloured in **blue**.

You can listen to the sample as many times as you need to.

Clarity

Very unclear Very clear

0 10 20 30 40 50 60 70 80 90 100

-

Harshness

Not harsh Very harsh

0 10 20 30 40 50 60 70 80 90 100

-

Distortion

Not distorted Very distorted

0 10 20 30 40 50 60 70 80 90 100

-

Spaciousness

Dead, not spacious Live, very spacious

0 10 20 30 40 50 60 70 80 90 100

-

Treble Strength

Not trebley Very trebley

0 10 20 30 40 50 60 70 80 90 100

-

Middle Strength

Not middley Very middley

0 10 20 30 40 50 60 70 80 90 100

-

Bass Strength

Not bassy Very bassy

0 10 20 30 40 50 60 70 80 90 100

-

Frequency Balance

Very bassy Balanced Very trebley

0 10 20 30 40 50 60 70 80 90 100

-

Overall Audio Quality

Very poor Very good

0 10 20 30 40 50 60 70 80 90 100

-

Feedback

Perceptual Attributes - Feedback on Use and Definitions

Thank you very much for listening to these new music samples.

The final part of this task is to revisit the perceptual attribute definitions, and provide feedback on how well you think these capture what the sensory panel discussed during the focus groups, and how easy it was to use the scales you developed.

To proceed, please click the arrow below.

Clarity

Please see the following proposed definition for the perceptual attribute that you as a group have labelled **clarity**:

Clarity refers to how well you can hear the different elements of the music, including being able to distinguish between the different sound sources, instruments, or voices in the music, and being able to hear the qualities that distinguish one sound source from another. Unclear music may sound indistinct, mushy, or muddy; clear music may sound clean, distinct, and transparent.

Short definition:

Clarity refers to how well you can hear and distinguish between the different instruments and elements within the music.

Scale endpoints: *very unclear*, and *very clear*

On the scale below, please indicate how well you feel that this definition encapsulates the overall meaning of your discussions as a group:

Not very well				Somewhat well				Extremely well		
0	10	20	30	40	50	60	70	80	90	100

-

Do you feel that the short definition presented above retains the essential meaning communicated in the full definition?

Yes

No

On the scale below, please indicate how easy you felt it was to use this attribute scale to rate the music samples:

Not very easy				Somewhat easy				Extremely easy		
0	10	20	30	40	50	60	70	80	90	100

-

Please feel free to include any comments or feedback or this definition in the space below:

Harshness

Please see the following proposed definition for the perceptual attribute that you as a group have labelled **harshness**:

Harshness refers to an emphasis or amplification of certain sound qualities (often in the treble frequencies or higher pitches) that can feel overwhelming, abrasive, painful, or

discomforting. Harsh sounds may sound piercing, screechy, shrill or sharp.

Short definition:

Harshness refers to an uncomfortable overemphasis of certain parts of the sound. It is most often heard in the treble resulting in a piercing, screechy or sharp sounds.

Scale endpoints: *not harsh*, and *very harsh*

On the scale below, please indicate how well you feel that this definition encapsulates the overall meaning of your discussions as a group:

Not very well				Somewhat well				Extremely well		
0	10	20	30	40	50	60	70	80	90	100

\-

Do you feel that the short definition presented above retains the essential meaning communicated in the full definition?

- Yes
- No

On the scale below, please indicate how easy you felt it was to use this attribute scale to rate the music samples:

Not very easy				Somewhat easy				Extremely easy		
0	10	20	30	40	50	60	70	80	90	100

\-

Please feel free to include any comments or feedback on this definition in the space below:

Distortion

Please see the following proposed definition for the perceptual attribute that you as a group have labelled **distortion**:

Distortion refers to a sense that the audio quality of the music contains elements that should not be there, that do not feel right, or that have appeared between the music's reproduction and your listening of it. These elements may include artefacts (e.g., noise, hiss, pops, crackles), or distortions to pitch (e.g., the pitches sound wrong compared to what you imagine was performed and recorded). No distortion may reflect a sense that the music is an authentic or accurate representation of what was performed and recorded, with no sense of pollution, interference, or distortion in the audio signal.

Short definition:

Distortion can be caused by artefacts that shouldn't be present e.g., noise, hiss, pops or crackles. It can also be caused by the pitches sounding wrong. Music with no distortion sounds like an authentic version of what was performed.

Scale endpoints: *not distorted*, and *very distorted*

On the scale below, please indicate how well you feel that this definition encapsulates the overall meaning of your discussions as a group:

Not very well		Somewhat well		Extremely well

0 10 20 30 40 50 60 70 80 90 100

-

Do you feel that the short definition presented above retains the essential meaning communicated in the full definition?

- Yes
- No

On the scale below, please indicate how easy you felt it was to use this attribute scale to rate the music samples:

Not very easy		Somewhat easy		Extremely easy

0 10 20 30 40 50 60 70 80 90 100

-

Please feel free to include any comments or feedback or this definition in the space below:

Spaciousness

Please see the following proposed definition for the perceptual attribute that you as a group have labelled **spaciousness**:

Imagine hitting a drum in two spaces: a small living room, and then a large cathedral. Whilst your action is the same, the sound produced can take less time or more time to return to silence, as it reverberates in a space.

Spaciousness refers to the perceived presence of these reverberations created by the space in which the music was performed. This may refer to how much you feel the music is 'coloured' by this space. A lack of spaciousness may mean that reverberations or a sense of space is not heard, with the opposite true for very spacious sound.

Short definition:

Spaciousness refers to how much you feel the music is 'coloured' by the performance space, and how much you can hear the reverberations and sense of space.

Scale endpoints: *Not spacious*, and *very spacious*

On the scale below, please indicate how well you feel that this definition encapsulates the overall meaning of your discussions as a group:

Not very well				Somewhat well				Extremely well		
0	10	20	30	40	50	60	70	80	90	100

-

Do you feel that the short definition presented above retains the essential meaning communicated in the full definition?

- Yes
- No

On the scale below, please indicate how easy you felt it was to use this attribute scale to rate the music samples:

Not very easy				Somewhat easy				Extremely easy		
0	10	20	30	40	50	60	70	80	90	100

-

Please feel free to include any comments or feedback or this definition in the space below:

Treble Strength

Please see the following proposed definition for the perceptual attribute that you as a group have labelled **treble strength**:

Treble strength refers to the perceived strength or prominence of sound qualities that are characterised by higher frequencies in the treble range, or similarly, sounds, instruments or voices with higher pitches.

Scale endpoints: *Not trebley*, and *very trebley*

On the scale below, please indicate how well you feel that this definition encapsulates the overall meaning of your discussions as a group:

Not very well				Somewhat well				Extremely well		
0	10	20	30	40	50	60	70	80	90	100

-

On the scale below, please indicate how easy you felt it was to use this attribute scale to rate the music samples:

Not very easy				Somewhat easy				Extremely easy		
0	10	20	30	40	50	60	70	80	90	100

-

Please feel free to include any comments or feedback or this definition in the space below:

Middle Strength

Please see the following proposed definition for the perceptual attribute that you as a group have labelled **middle strength**:

Middle strength refers to the perceived strength or prominence of sound qualities that are characterised by middle frequencies found between bass and treble ranges, or similarly, sounds, instruments or voices that pitches perceived as being between lower and higher pitches.

Scale endpoints: *Not middley*, and *very middley*

On the scale below, please indicate how well you feel that this definition encapsulates the overall meaning of your discussions as a group:

Not very well Somewhat well Extremely well

0 10 20 30 40 50 60 70 80 90 100

-

On the scale below, please indicate how easy you felt it was to use this attribute scale to rate the music samples:

Not very easy Somewhat easy Extremely easy

0 10 20 30 40 50 60 70 80 90 100

-

Please feel free to include any comments or feedback or this definition in the space below:

Bass Strength

Please see the following proposed definition for the perceptual attribute that you as a group have labelled **bass strength**:

Bass strength refers to the perceived strength or prominence of sound qualities that are characterised by lower frequencies in the bass range, or similarly, sounds, instruments or voices with lower pitches.

Scale endpoints: *Not bassy*, and *very bassy*

On the scale below, please indicate how well you feel that this definition encapsulates the overall meaning of your discussions as a group:

Not very well				Somewhat well				Extremely well		
0	10	20	30	40	50	60	70	80	90	100

\-

On the scale below, please indicate how easy you felt it was to use this attribute scale to rate the music samples:

Not very easy				Somewhat easy				Extremely easy		
0	10	20	30	40	50	60	70	80	90	100

\-

Please feel free to include any comments or feedback or this definition in the space below:

Frequency Balance

Please see the following proposed definition for the perceptual attribute of **frequency balance**:

Frequency balance refers to the perceived, relative balance between treble (or higher pitches of sound) and bass (or lower pitches of sound) in the audio. Audio described as more bassy would be characterised as having stronger or more prominent bass frequencies and pitches in comparison to treble frequencies and pitches, with the opposite true for audio described as more trebley. The middle point of this scale indicates a perceived balance between bass and treble.

Short definition:

Frequency balance refers to the perceived balance between treble (or higher pitch) and bass (or lower pitch) sounds.

Scale endpoints: *very bassy*, and *very trebley*
Scale midpoint: *balanced*

On the scale below, please indicate how well you feel that this definition encapsulates the overall meaning of your discussions as a group:

Not very well				Somewhat well				Extremely well		
0	10	20	30	40	50	60	70	80	90	100

-

Do you feel that the short definition presented above retains the essential meaning communicated in the full definition?

- Yes
- No

On the scale below, please indicate how easy you felt it was to use this attribute scale to rate the music samples:

Not very easy				Somewhat easy				Extremely easy		
0	10	20	30	40	50	60	70	80	90	100

-

Please feel free to include any comments or feedback or this definition in the space below:

Overall Audio Quality

Please see the following proposed definition for **overall audio quality**:

Perceived audio quality results from judgments of the sound of the music, in relation to a person's expectations of how the music should ideally sound to them.

Imagine listening to a piece of music in two different ways: listening through a cheap mobile phone, and then listening through high quality loudspeakers. The music is fundamentally same in both cases, but the audio quality is very different.

Scale endpoints: *very poor,* and *very good*

On the scale below, please indicate how well you feel that this definition encapsulates the overall meaning of your discussions as a group:

Not very well				Somewhat well				Extremely well		
0	10	20	30	40	50	60	70	80	90	100

-

On the scale below, please indicate how easy you felt it was to use this attribute scale to rate the music samples:

Not very easy				Somewhat easy				Extremely easy		
0	10	20	30	40	50	60	70	80	90	100

-

Please feel free to include any comments or feedback or this definition in the space below:

General Feedback

Thank you very much once again for completing this follow-up task. You have now reached the end of this task.

If there is any more general feedback about the sensory panel, perceptual attributes, or other aspects, please do feel welcome to write something in the space provided below!

Powered by Qualtrics
